# AGV navigation in complex environments: A cooperative trajectory optimization strategy based on SMF-MPC

**DOI:** 10.1371/journal.pone.0355960

**Published:** 2026-08-26

**Authors:** Zonghe Ding, Ling Chen, Jintao Wang, Qi Qu, Liming Chang, Yajing Cheng

**Affiliations:** 1 School of Electrical and Information Engineering, Wanjiang University of Technology, Ma’anshan, China; 2 College of Artifcial Intelligence and Automation, Hohai University, Changzhou, China; Vignan’s Institute of Information Technology, INDIA

## Abstract

This paper addresses the navigation problem of automated guided vehicles (AGVs) in complex environments by proposing a collaborative trajectory optimization strategy based on the Set Membership Filter (SMF) and Model Predictive Control (MPC). In contrast, the traditional probability distribution-based navigation method, UKF-MPC, often fails to provide a strict safety margin when dealing with UBB noise, thereby increasing the risk of navigation failure. Therefore, this paper proposes a set-membership filter algorithm that constructs a minimum enclosing ellipsoid to enclose the system’s true state in real time and incorporates the geometric characteristics of this ellipsoid as a time-varying penalty term into the MPC optimization objective, thereby achieving dynamic risk avoidance. Finally, comparative experiments under three different types of environmental noise were conducted using MATLAB. The MATLAB simulation results indicate that, under complex environmental disturbances, the SMF-MPC control strategy exhibits a higher navigation success rate and greater robustness than both the traditional UKF-MPC control strategy and the Robust MPC control strategy.

## 1. Introduction

With the deep integration of Industry 4.0 and smart logistics, AGVs have become indispensable core equipment in smart manufacturing systems, warehousing and logistics, and automated terminals [1,2]. In complex dynamic environments (such as high-density warehouses and human-robot collaborative workshops), AGVs must not only possess efficient path-tracking capabilities but also ensure extremely high navigation safety and robustness in the face of environmental disturbances [3,4]. However, sensor measurement noise, mechanical transmission errors, and random disturbances caused by uneven surfaces—which are prevalent in real-world industrial settings—pose significant challenges to AGV positioning accuracy and trajectory control [5,6].

Traditional AGV navigation and control frameworks largely rely on stochastic filtering algorithms based on probability distributions, such as the Extended Kalman Filter (EKF) and the Unbiased Kalman Filter (UKF) [7,8]. These methods perform well under the assumption that noise follows a Gaussian distribution [9]. However, in real-world operating conditions, environmental disturbances often exhibit unstructured and time-varying characteristics, manifesting as UBB noise with unknown boundaries rather than an ideal Gaussian distribution [10,11]. In such scenarios, probability-based filtering methods often fail to provide strict state estimation bounds, causing the system to potentially exceed its predefined range under extreme disturbances, thereby increasing the risk of collisions or even leading to navigation failure [12,13].

MPC has been widely applied in the field of AGV motion control due to its ability to effectively handle system constraints and multi-objective optimization. In [14], the authors focused on solving the robust collision-avoidance formation navigation problem for a class of multi-unmanned surface vehicles and developed a novel controller based on distributed MPC to achieve collision-free formation navigation. In [15], a convex optimization model was derived to optimize dynamic feedback laws for linear systems subject to polyhedral uncertainty constraints, and a computationally efficient robust model predictive control law was defined.In recent years, researchers have attempted to combine UKF with MPC to enhance the controller’s robustness against disturbances by predicting the probability distribution of the state [16]. In [17], a highly nonlinear dynamic environment was considered; since simple fixed-parameter linear controllers were unable to cope with the constantly changing dynamics and the various disturbance sources affecting the solar farm, a model predictive control strategy based on the UKF was designed and successfully applied in this field.However, when dealing with safety-critical tasks subject to strict hard constraints, such schemes struggle to mathematically guarantee that the system state remains within the safety domain at all times, due to the lack of an accurate description of state uncertainty [18,19].

As a deterministic estimation method for handling UBB noise, the Set-Membership Filtering algorithm offers a new approach for navigation tasks with high safety requirements [20,21]. By constructing a minimal set that encloses the true state, SMF describes state uncertainty and provides the controller with a well-defined feasible state space. In [22], a set-membership filter is designed for all permissible unknown but bounded noise, such that the ellipsoidal set containing all possible states can be determined using a convex optimization method subject to probabilistic constraints. In [23], a new data-selective adaptive filtering algorithm—the Set-Membership Affine Projection (SM-AP) algorithm—was proposed. This algorithm demonstrates excellent performance in terms of convergence rate, final error, and reduced computational complexity, particularly when processing colored input signals. In [24], the distributed set-membership filtering problem was investigated for a class of time-varying multi-rate systems in sensor networks employing communication protocols. A set of distributed filters was designed, and the effectiveness of the proposed protocol-based distributed set-membership filtering algorithm was verified through numerical simulations. The aforementioned studies demonstrate that, compared to probabilistic filtering, SMF offers significant advantages in terms of robustness. Therefore, exploring a deep cooperative algorithm that integrates SMF and MPC, which transforms the geometric characteristics of state uncertainty into dynamic constraints for the control law, represents a key direction for enhancing the reliability of AGV navigation in complex environments [25,26].

In response to the aforementioned challenges, this paper proposes a collaborative trajectory optimization strategy based on SMF-MPC. The main contributions of this work are as follows:

1. Kinematics and Noise Modeling: A kinematic model of the AGV that accounts for nonlinear characteristics was developed, and a refined UBB noise model was introduced to address disturbances in complex environments [27,28];
2. SMF-MPC Collaboration Framework: Existing SMF-MPC research has primarily focused on trajectory tracking problems. These studies typically treat the SMF’s estimated ellipsoid merely as a “more accurate single-point estimate,” without actively incorporating the ellipsoid’s geometric information into the MPC optimization objective. This paper provides a detailed derivation of the time update and measurement update algorithms for nonlinear ellipsoid membership filtering. It innovatively introduces the geometric features of the real-time generated minimum enclosing ellipsoid as a time-varying penalty term into the MPC objective function, thereby formulating an MPC optimization problem that incorporates ellipsoid membership constraints;
3. Multi-scenario experimental validation: Under three types of environmental noise interference, the proposed SMF-MPC strategy was thoroughly compared with the traditional UKF-MPC. The experimental results demonstrate that this strategy exhibits higher navigation success rates and greater robustness under complex environmental interference [29–31].

## 2. Development of a system model

### 2.1. Model development

**Fig 1:**
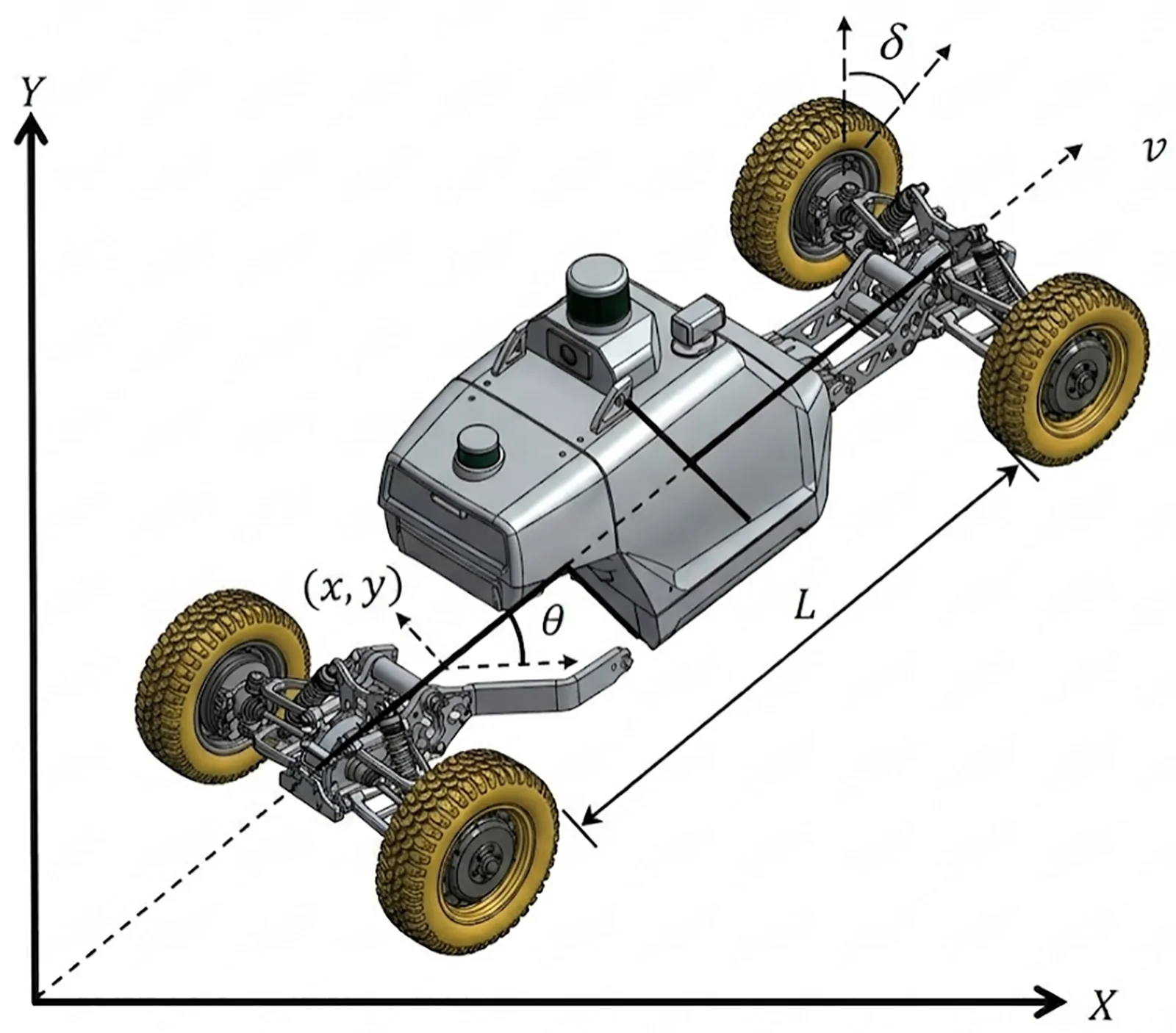
Four-wheel differential drive AGV.

In SMF-based state estimation, system modeling is a critical component. Appropriate kinematic and observation models not only determine the estimation accuracy of the filter but also influence the formulation of constraints in subsequent MPC optimization. This paper focuses on a typical four-wheel differential-drive AGV, whose motion in a planar environment can be modeled using incomplete constrained kinematic equations, as shown in Fig 1.

When a differential-drive AGV moves on a plane, it exhibits the following characteristics: The AGV controls its linear and angular velocities by adjusting the speeds of its left and right wheels; due to incomplete constraints, its lateral velocity is uncontrollable, and its lateral displacement is determined solely by the heading angle and linear velocity; since control errors, tire slip, and environmental disturbances are present during actual operation, process noise is explicitly incorporated into the model.


$$\begin{array}{c} X={\left[\begin{array}{cccc} @cccc@x & y & \theta & v \end{array}\right]}^{T} \end{array}$$ (1)

Where: $\left(\begin{array}{cc} @cc@x & y \end{array}\right)$ represents the AGV’s position in the global coordinate system, θ represents the heading angle, and v represents the linear velocity.

Using simplified AGV kinematic equations, the continuous-time state equations are as follows:


$$\begin{array}{c} \dot{X}=\left[\begin{array}{c} @c@\dot{x}\\ \dot{y}\\ \dot{\theta }\\ \dot{v} \end{array}\right]=\left[\begin{array}{c} @c@vcos\theta \\ vsin\theta \\ \frac{vtan\delta }{L}\\ a \end{array}\right] \end{array}$$ (2)

The control inputs are:


$$\begin{array}{c} u={\left[\begin{array}{cc} @cc@\delta & a \end{array}\right]}^{T} \end{array}$$ (3)

Using the Euler method to discretize the time with a sampling period $T_{s}$, we obtain:


$$\begin{array}{c} X_{k+1}=f\left(X_{k},u_{k}\right)+w_{k} \end{array}$$ (4)

The nonlinear function $f:{\text{R}}^{\text{4}}\times {\text{R}}^{\text{2}}\to {\text{R}}^{\text{4}}$ is defined as:


$$\begin{array}{c} f\left(X_{k},u_{k}\right)=\left[\begin{array}{c} @c@x_{k}+T_{s}v_{k}\;cos\theta_{k}\\ y_{k}+T_{s}v_{k}\;sin\theta_{k}\\ \frac{\theta_{k}+T_{s}v_{k}\;tan\delta_{k}}{L}\\ v_{k}+T_{s}a_{k} \end{array}\right] \end{array}$$ (5)

The observational equation is:


$$\begin{array}{c} Y_{k}=h\left(X_{k}\right)+v_{k} \end{array}$$ (6)

To simplify the problem, this paper adopts a linearly observable model that directly observes all states:


$$\begin{array}{c} h\left(X_{k}\right)=HX_{k}=\left[\begin{array}{cccc} @cccc@1 & 0 & 0 & 0\\ 0 & 1 & 0 & 0\\ 0 & 0 & 1 & 0\\ 0 & 0 & 0 & 1 \end{array}\right]X_{k} \end{array}$$ (7)

where: $\delta_{k}$ is the front-wheel steering angle, $a_{k}$ is the acceleration,L is the wheelbase, $w_{k}\in {\text{R}}^{\text{4}}$ is the process noise, and $v_{k}\in {\text{R}}^{\text{4}}$ is the measurement noise.

### 2.2. UBB Noise assumption

All noise sources in actual AGV systems are physically bounded. These physical boundaries are objectively present, measurable, and tied to specific components, whereas the specific statistical distribution of the noise may vary with factors such as time, temperature, and aging. The classical Kalman filter framework implicitly assumes that the noise follows a Gaussian distribution; however, in industrial environments, a more natural modeling approach is to treat the statistical distribution as unknown while the physical boundaries are known. AGV navigation is a safety-critical task. Probabilistic methods (such as UKF) can only provide a guarantee of “high-probability safety” and cannot rule out low-probability but catastrophic events.

**Assumption 1 (UBB noise)**. The process noise $w_{k}$ and measurement noise $v_{k}$ belong to a known compact ellipsoidal set:


$$\begin{array}{c} w_{k}\in W=\left\{\omega :\omega^{T}Q^{-\text{1}}\omega \leq \text{1}\right\} \end{array}$$ (8)

$$\begin{array}{c} v_{k}\in V=\left\{v:v^{T}R^{-\text{1}}v\leq \text{1}\right\} \end{array}$$ (9)

In particular, Q≻0 and R≻0 are known shape matrices that describe the amplitude boundaries of the noise, but do not assume any specific probability distribution for the noise.

The process noise boundary matrix Q for AGVs is primarily caused by model mismatch, while the measurement noise boundary matrix R originates from the sensors, as follows:


$$\begin{array}{c} {\text{w}}_{k}=[w_{x},w_{y},w_{\theta },w_{v}{]}^{T} \end{array}$$ (10)

$$\begin{array}{c} {\text{v}}_{k}=[v_{x},v_{y},v_{\theta },v_{v}{]}^{T} \end{array}$$ (11)

$w_{x},w_{y}$ is determined by the maximum unmodeled acceleration; $w_{\theta }$ is determined by the maximum angular velocity fluctuation; $w_{v}$ is determined by the maximum acceleration; $v_{x},v_{y}$ are determined by the maximum position measurement error, $v_{\theta }$ is determined by the maximum heading measurement noise, and $v_{v}$ is determined by the maximum velocity measurement noise.

Taking into account the process noise and measurement noise described above, we obtain the following boundary matrix:


$$\begin{array}{c} Q=\;\text{diag}(q_{x}^{\text{2}},q_{y}^{\text{2}},q_{\theta }^{\text{2}},q_{v}^{\text{2}}) \end{array}$$ (12)

$$\begin{array}{c} R=\;\text{diag}(r_{x}^{\text{2}},r_{y}^{\text{2}},r_{\theta }^{\text{2}},r_{v}^{\text{2}}) \end{array}$$ (13)

The choice of the boundary matrix here directly determines the compactness of the ellipsoid estimated by the SMF method and the reliability of the inclusion guarantee. Specifically, the shape matrix is chosen to be a diagonal matrix whose diagonal elements are equal to the square of the maximum amplitude of each component. This choice ensures that the actual noise vector lies inside the ellipsoid and, when the components are independent of one another, that the ellipsoid precisely encloses the rectangular region of the noise.

## 3. Design of set-membership filter algorithm

The objective of set-member filtering is to recursively estimate the feasible set $\chi_{k}$ for the state. It assumes that the noise is unknown but bounded, and continuously adjusts the size and orientation of the ellipsoid based on observed data so that, while satisfying the constraints, the true state $\chi_{k}$ satisfies$X_{k}\in \chi_{k}$. In set-member filtering, $\chi_{k}$ is approximated by an ellipsoid:


$$\begin{array}{c} \varepsilon \left({\hat{X}}_{k},P_{k}\right)=\left\{X:(X-{\hat{X}}_{k}{)}^{T}P_{k}^{-1}\left(X-{\hat{X}}_{k}\right)\leq 1\right\} \end{array}$$ (14)

Where: ${\hat{X}}_{k}$ is the center of the ellipsoid, and $P_{k}≻\;\text{0}$ is the shape matrix, which determines the size and orientation of the ellipsoid. Assuming that the feasible set at time k is $\varepsilon \left({\hat{X}}_{k},P_{k}\right)$, we consider the nonlinear system (2) together with the UBB process noise (8) and (9) to further compute the predicted feasible set.

### 3.1. Linearization of nonlinear functions

Perform a Taylor expansion of the nonlinear function f at ${\hat{X}}_{k}$, retaining the first-order term:


$$\begin{array}{c} f\left(X_{k},u_{k}\right)=f\left({\hat{X}}_{k},u_{k}\right)+F_{k}\left(X_{k}-{\hat{X}}_{k}\right)+e_{f} \end{array}$$ (15)

Where: $e_{f}$ is the linearized residual term, and $F_{k}=\frac{\partial f}{\partial X}{|}_{{\hat{X}}_{k}}$ is the Jacobian matrix.

The specific form of the Jacobian matrix $F_{k}$ is:


$$\begin{array}{c} F_{k}=\left[\begin{array}{cccc} @cccc@1 & 0 & -T_{s}v_{k}sin\theta_{k} & T_{s}cos\theta_{k}\\ 0 & 1 & T_{s}v_{k}cos\theta_{k} & T_{s}sin\theta_{k}\\ 0 & 0 & 1 & T_{s}tan\delta_{k}/L\\ 0 & 0 & 0 & 1 \end{array}\right] \end{array}$$ (16)

In the time update of the SMF, the linearization residual term ${\text{e}}_{f}$ of the nonlinear function f must be bounded by the ellipsoid $E(\text{0},{𝐄}_{f})$. The Lipschitz constant provides the theoretical basis for this bound.

**Lemma 1 (Ellipsoidal Bounds for the Taylor Residual).** If $f_{i}$ has a Lipschitz-continuous gradient on $E({\hat{𝐱}}_{k},{𝐏}_{k})$, then the linearized residual satisfies:


$$\begin{array}{c} |e_{f,i}|\leq \frac{\text{1}}{\text{2}}L_{i}\cdot ∥𝐱-{\hat{𝐱}}_{k}{∥}^{\text{2}} \end{array}$$ (17)

Where: $L_{i}$ is the Lipschitz constant of $\nabla f_{i}$.

Furthermore, using $∥𝐱-{\hat{𝐱}}_{k}{∥}^{\text{2}}\leq \lambda_{\text{max}}({𝐏}_{k}^{-\text{1}})$, we obtain:


$$\begin{array}{c} |e_{f,i}|\leq \frac{\text{1}}{\text{2}}L_{i}\cdot \lambda_{\text{max}}({𝐏}_{k}^{-\text{1}})≜{\bar{e}}_{f,i} \end{array}$$ (18)

Therefore, ${𝐄}_{f}=\text{diag}({\bar{e}}_{f,\text{1}}^{\text{2}},\ldots ,{\bar{e}}_{f,n}^{\text{2}})$.

For the AGV model, the Lipschitz constants of the components of $f=[f_{\text{1}},f_{\text{2}},f_{\text{3}},f_{\text{4}}{]}^{T}$ are as follows:


$$\begin{array}{cccc} f_{\text{1}} & =x+T_{s}vcos\theta ,\; & f_{\text{2}} & =y+T_{s}vsin\theta ,\\ f_{\text{3}} & =\theta +T_{s}vtan\delta /L,\; & f_{\text{4}} & =v+T_{s}a \end{array}$$ (19)

The gradients of the components of $𝐱=[x,y,\theta ,v{]}^{T}$ are:


$$\begin{array}{cc} \nabla f_{\text{1}} & =[\text{1},\;\text{0},\;-T_{s}v\text{sin}\theta ,\;T_{s}\text{cos}\theta {]}^{T}\\ \nabla f_{\text{2}} & =[\text{0},\;\text{1},\;T_{s}v\text{cos}\theta ,\;T_{s}\text{sin}\theta {]}^{T}\\ \nabla f_{\text{3}} & =[\text{0},\;\text{0},\;\text{1},\;T_{s}\text{tan}\delta /L{]}^{T}\\ \nabla f_{\text{4}} & =[\text{0},\;\text{0},\;\text{0},\;\text{1}{]}^{T} \end{array}$$ (20)

An upper bound on the gradient norm is:


$$\begin{array}{cc} ∥\nabla f_{\text{1}}∥ & \leq \sqrt{\text{1}+(T_{s}v_{max}{)}^{\text{2}}+T_{s}^{\text{2}}}\approx \text{1}.\text{012}\\ ∥\nabla f_{\text{2}}∥ & \leq \sqrt{\text{1}+(T_{s}v_{max}{)}^{\text{2}}+T_{s}^{\text{2}}}\approx \text{1}.\text{012}\\ ∥\nabla f_{\text{3}}∥ & \leq \sqrt{\text{1}+(T_{s}\text{tan}\delta_{max}/L{)}^{\text{2}}}\approx \text{1}.\text{002}\\ ∥\nabla f_{\text{4}}∥ & =\text{1} \end{array}$$ (21)

Where: $T_{s}=\text{0}.\text{1s},\;v_{max}=\text{1}.\text{5m}/\text{s},\;\delta_{max}=\text{0}.\text{5rad}$.

The Lipschitz constant is defined as an upper bound on the gradient norm:


$$\begin{array}{c} L_{\text{1}}=L_{\text{2}}=\text{1}.\text{012},\;L_{\text{3}}=\text{1}.\text{002},\;L_{\text{4}}=\text{1} \end{array}$$ (22)

Based on Lemma 1 above, we obtain:


$$\begin{array}{c} E_{f}=diag\left(\left[\frac{L_{\text{1}}^{\text{2}}}{\text{4}},\frac{L_{\text{2}}^{\text{2}}}{\text{4}},\frac{L_{\text{3}}^{\text{2}}}{\text{4}},\frac{L_{\text{4}}^{\text{2}}}{\text{4}}\right]\right) \end{array}$$ (23)

Where: $L_{i}$ is the Lipschitz constant of $f_{i}$ at $\varepsilon \left({\hat{X}}_{k},P_{k}\right)$.

**Lemma 2 (Minkowski sum of ellipsoids).** For any two ellipsoids ε(0,A) and ε(0,\hspace{0.17em}B), their vector sum lies within the ellipsoid:


$$\begin{array}{c} \varepsilon \left(0,\text{\textbackslash{}hspace\{0.17em}\}A\right)+\varepsilon \left(0,B\right)\subseteq \varepsilon \left(0,\left(1+p\right)A+\left(1+p^{-1}\right)B\right),\forall p>0 \end{array}$$ (24)

**Proof of Lemma 2**. For any a∈ε(0,A),b∈ε(0,B), there exist $a^{T}A^{-1}a\leq 1,b^{T}B^{-1}b\leq 1$. By Cauchy’s inequality, for any p>0:


$$\begin{array}{c} {\left(a+b\right)}^{T}{\left[\left(1+p\right)A+\left(1+p^{-1}\right)B\right]}^{-1}\left(a+b\right)\leq a^{T}A^{-1}a+b^{T}B^{-1}b\leq 2 \end{array}$$ (25)

From Formula (24), we can obtain $\frac{\text{1}}{\sqrt{\text{2}}}(a+b)\in \left(0,\left(1+p\right)A+\left(1+P^{-1}\right)B\right)$. It can also be expressed as $a+b\in \varepsilon \left(0,2\left[\left(1+p\right)A+\left(1+p^{-1}\right)B\right]\right)$. By adjusting the coefficients, we obtain the standard form. □.

**Theorem 1.** If $X_{k}\in \varepsilon \left({\hat{X}}_{k},P_{k}\right),w_{k}\in \left(0,Q\right)$, then we further predict that state $X_{k+1|k}$ is contained within ellipsoid $\varepsilon \left({\hat{X}}_{k+1|k},P_{k+1|k}\right)$.


$$\begin{array}{c} {\hat{X}}_{k+1|k}=f\left({\hat{X}}_{k},u_{k}\right) \end{array}$$ (26)

$$\begin{array}{c} P_{k+1|k}=\left(1+p\right)F_{k}P_{k}F_{k}^{T}+\left(1+p^{-1}\right)\left(E_{f}+Q\right) \end{array}$$ (27)

Parameter p>0 is selected through optimization by minimizing the ellipsoid volume:


$$\begin{array}{c} p^{*}=arg\underset{p>0}{min}det\left(P_{k+1|k}\left(p\right)\right) \end{array}$$ (28)

**Where:** ${\text{P}}_{k+\text{1}|k}(p)=(\text{1}+p){\text{M}}_{\text{1}}+(\text{1}+p^{-\text{1}}){\text{M}}_{\text{2}}$, ${\text{M}}_{\text{1}}={\text{F}}_{k}{\text{P}}_{k}{\text{F}}_{k}^{T}$, ${\text{M}}_{\text{2}}={\text{E}}_{f}+Q$, $p^{*}\in \left[\begin{array}{cc} @cc@0.1 & 2.0 \end{array}\right]$.

**Proof of Theorem 1**. From the properties of ellipsoidal linear transformations, we can see that $F_{k}\varepsilon \left({\hat{X}}_{k},P_{k}\right)\subseteq \varepsilon \left(F_{k}{\hat{X}}_{k},F_{k}P_{k}F_{k}^{T}\right)$. The vector sum of the UBB noise ellipsoid ε(0,Q) and the linearization error ellipsoid $\varepsilon \left(0,E_{f}\right)$ is $\varepsilon \left(0,E_{f}\right)+\varepsilon \left(0,Q\right)$. By Lemma 1, for any p>0, we have.


$$\begin{array}{c} \varepsilon \left(0,E_{f}\right)+\varepsilon \left(0,Q\right)\subseteq \varepsilon \left(0,\left(1+p\right)E_{f}+\left(1+p^{-1}\right)Q\right) \end{array}$$ (29)

Since the linearization error and process noise are independent of each other, the prediction set is the sum of the two ellipsoids mentioned above:


$$\begin{array}{c} \chi_{k+1|k}=F_{k}\varepsilon \left({\hat{X}}_{k},P_{k}\right)+\left[\varepsilon \left(0,E_{f}\right)+\varepsilon \left(0,Q\right)\right] \end{array}$$ (30)

Therefore, formula (29) verifies that the predicted state lies within the ellipsoid.

### 3.2. Measurement update

Suppose the prior feasible set is $\varepsilon \left({\hat{X}}_{k+1|k},P_{k+1|k}\right)$; after obtaining a new observation $Y_{k+1}$, we need to further compute the posterior feasible set. Expand the observation function h at ${\hat{X}}_{k+1|k}$:


$$\begin{array}{c} h\left(x\right)=h\left({\hat{X}}_{k+1|k}\right)+H_{k+1}\left(X-{\hat{X}}_{k+1|k}\right)+e_{h} \end{array}$$ (31)

Where: $H_{k+1}=\frac{\partial h}{\partial X}{|}_{{\hat{X}}_{k+1|k}},e_{h}$is the linearized residual term, enclosed by the ellipsoid $\varepsilon \left(0,E_{h}\right)$. For a linear observation model, $H_{k+1}=H$ is a constant matrix, $e_{h}=0$, and therefore $E_{h}=0$.

Create an observation strip. the observation information defines the strip area:


$$\begin{array}{c} S_{k+1}=\left\{X:{\left(Y_{k+1}-h\left(x\right)\right)}^{T}R^{-1}\left(Y_{k+1}-h\left(x\right)\right)\leq 1\right\} \end{array}$$ (32)

Taking linearization errors into account, this can be approximated as an expansion band:


$$\begin{array}{c} {\tilde{S}}_{k+1}=\left\{X:∥Y_{k+1}-h\left({\hat{X}}_{k+1|k}\right)-H_{k+1}(X-{\hat{X}}_{k+1|k})|{|}_{R^{-1}}\leq 1+\delta \right\} \end{array}$$ (33)

Where: δ is the expansion factor used to compensate for linearization errors, and may be set to an upper bound of $\delta =\sqrt{e_{h}^{T}R^{-1}e_{h}}$. For linear observations, δ=0 applies.

**Theorem 2.** Predict that the intersection of ellipsoid $\varepsilon \left({\hat{X}}_{k+1|k},P_{k+1|k}\right)$ and strip ${\tilde{S}}_{k+1}$ is contained within ellipsoid $\varepsilon \left({\hat{X}}_{k+1|k},P_{k+1}\right)$, where:


$$\begin{array}{c} {\hat{X}}_{k+1}={\hat{X}}_{k+1|k}+\lambda P_{k+1|k}H_{k+1}^{T}\Delta^{-1}v \end{array}$$ (34)

$$\begin{array}{c} P_{k+1}=\beta \left(P_{k+1|k}-\lambda P_{k+1|k}H_{k+1}^{T}\Delta^{-1}H_{k+1}P_{k+1|k}\right) \end{array}$$ (35)

the remaining terms are as follows:


$$\begin{array}{c} v=Y_{k+1}-h\left({\hat{X}}_{k+1|k}\right) \end{array}$$

$$\begin{array}{c} \Delta =H_{k+1}P_{k+1|k}H_{k+1}^{T}+\frac{1+\delta }{\lambda }R \end{array}$$

$$\begin{array}{c} \beta =1+\delta -\lambda v^{T}\Delta^{-1}v \end{array}$$

In the measurement update, λ is determined by minimizing the posterior ellipsoid volume:


$$\begin{array}{c} \lambda^{*}=\text{arg}\underset{\lambda \in [\text{0},\text{1}]}{\text{min}}\text{det}({\text{P}}_{\text{k}+\text{1}}\left(\lambda )\right) \end{array}$$ (36)

Where: ${\text{P}}_{k+\text{1}}(\lambda )=\beta (\lambda )\left({\text{P}}_{k+\text{1}|k}-\lambda {\text{P}}_{k+\text{1}|k}{\text{H}}^{T}\Delta (\lambda {)}^{-\text{1}}\text{H}{\text{P}}_{k+\text{1}|k}\right)$,$\lambda^{*}\in \left[\begin{array}{cc} @cc@0.05 & 0.95 \end{array}\right]$

Constraints: To ensure that ${\text{P}}_{k+\text{1}}≻\;\text{0}$, the following conditions must be satisfied:${\text{P}}_{k+\text{1}|k}-\lambda {\text{P}}_{k+\text{1}|k}{\text{H}}^{T}\Delta (\lambda {)}^{-\text{1}}\text{H}{\text{P}}_{k+\text{1}|k}≻\;\text{0}$. β(λ)>0 is equivalent to $\text{1}+\delta -\lambda \nu^{T}\Delta (\lambda {)}^{-\text{1}}\nu >\text{0}$.

**Proof of Theorem 2.** The minimum-volume enveloping ellipsoid where an ellipsoid intersects a strip can be obtained by solving the following optimization problem:


$$\begin{array}{c} \underset{\hat{X},P}{min}det\left(P\right)\begin{array}{c} s.t. \end{array}\varepsilon \left({\hat{X}}_{k+1|k},P_{k+1|k}\right)⋂{\tilde{S}}_{k+1}\subseteq \varepsilon \left(\hat{X},P\right) \end{array}$$ (37)

For any A that satisfies the two constraints, we have:


$$\begin{array}{c} {\left(X-{\hat{X}}_{+}\right)}^{T}P_{+}^{-1}\left(X-{\hat{X}}_{+}\right)\leq 1 \end{array}$$ (38)

$$\begin{array}{c} {\left(X-{\hat{X}}_{+}\right)}^{T}P_{+}^{-1}\left(X-{\hat{X}}_{+}\right)=\frac{\text{1}}{\beta_{p}}\left[\begin{array}{c} @l@{\left(X-\hat{X}\right)}^{T}P^{-1}\left(X-\hat{X}\right)\\ +\frac{\text{1}}{\delta^{\text{2}}}{\left(v-H\left(X-\hat{X}\right)\right)}^{T}R^{-1}\left(v-H\left(X-\hat{X}\right)\right)\\ -\frac{\text{1}}{\delta^{\text{2}}}v^{T}\Delta_{p}^{-1}v \end{array}\right] \end{array}$$ (39)

Two constraints:


$$\begin{array}{c} {\left(X-\hat{X}\right)}^{T}P^{-1}\left(X-\hat{X}\right)\leq 1 \end{array}$$ (40)

$$\begin{array}{c} {\left(v-H\left(X-\hat{X}\right)\right)}^{T}R^{-1}\left(v-H\left(X-\hat{X}\right)\right)\leq {\overset{―}{\delta }}^{\text{2}} \end{array}$$ (41)

Substituting (38) gives:


$$\begin{array}{c} \left(X-{\hat{X}}_{+}\right)P_{+}^{-1}\left(X-{\hat{X}}_{+}\right)\leq \frac{\text{1}}{\beta_{c}}\left(1+\lambda -\frac{\text{1}}{{\overset{―}{\delta }}^{\text{2}}}v^{T}\Delta_{p}^{-1}v\right) \end{array}$$ (42)

By the definition of $\beta_{p}$. $\beta_{p}={\overset{―}{\delta }}^{\text{2}}-\lambda v^{T}\Delta_{P}^{-1}v,{\overset{―}{\delta }}^{\text{2}}\geq \lambda v^{T}\Delta_{P}^{-1}v$ (otherwise the intersection is empty), therefore:


$$\begin{array}{c} {\left(X-{\hat{X}}_{+}\right)}^{T}P_{+}^{-1}\left(X-{\hat{X}}_{+}\right)\leq 1 \end{array}$$ (43)

Parameter λ is determined by minimizing $det\left(P_{+}\right)$, by the matrix determinant lemma:


$$\begin{array}{c} det\left(P_{+}\right)={\beta_{p}}^{nX}det\left(P\right)det\left(I-\lambda P^{\frac{\text{1}}{\text{2}}}H^{T}\Delta_{p}^{-1}HP^{\frac{\text{1}}{\text{2}}}\right) \end{array}$$ (44)

Substituting the expression for $\Delta_{p}$ yields an expression for $det\left(P_{+}\right)$ in terms of λ. By solving for $\frac{d}{d\lambda }det\left(P_{+}\right)=0$, we obtain the optimal λ. The analytical solution is as follows:


$$\begin{array}{c} {\hat{X}}_{+}=\hat{X}+\lambda PH^{T}\Delta_{p}^{-1}v \end{array}$$ (45)

$$\begin{array}{c} P_{+}=\beta_{p}\left(P-\lambda PH^{T}\Delta_{p}^{-1}HP\right) \end{array}$$ (46)

Where:


$$\begin{array}{c} \Delta_{p}=HPH^{T}+\frac{{\overset{―}{\delta }}^{\text{2}}}{\lambda }R \end{array}$$

$$\begin{array}{c} \beta_{p}={\overset{―}{\delta }}^{\text{2}}-\lambda v^{T}\Delta_{P}^{-1}v \end{array}$$

Parameter $\lambda \in \left[\begin{array}{cc} @cc@0 & 1 \end{array}\right]$ is determined by minimizing $det\left(P_{+}\right)$; this result is in full agreement with Theorem 2. □

## 4. Design of the SMF-MPC obstacle avoidance algorithm

Model Predictive Control has become the mainstream method for path planning due to its ability to handle constraints and the forward-looking nature of rolling optimization. However, traditional MPC methods assume that the system state is fully known or accurately estimated through filtering, which poses two major challenges in practical applications: first, the problem of dynamic obstacle avoidance caused by environmental complexity; and second, estimation errors resulting from perception uncertainty. The set-membership filtering method adopted in this paper provides a deterministic framework for state estimation under UBB noise. Unlike traditional Kalman filtering, SMF does not assume a specific noise distribution; instead, it computes a minimal ellipsoid set containing the true state through recursive calculations, thereby providing a deterministic uncertainty boundary. By combining SMF with MPC, state uncertainty can be accounted for during the control process, enabling safer obstacle-avoidance navigation.

**Fig 2:**
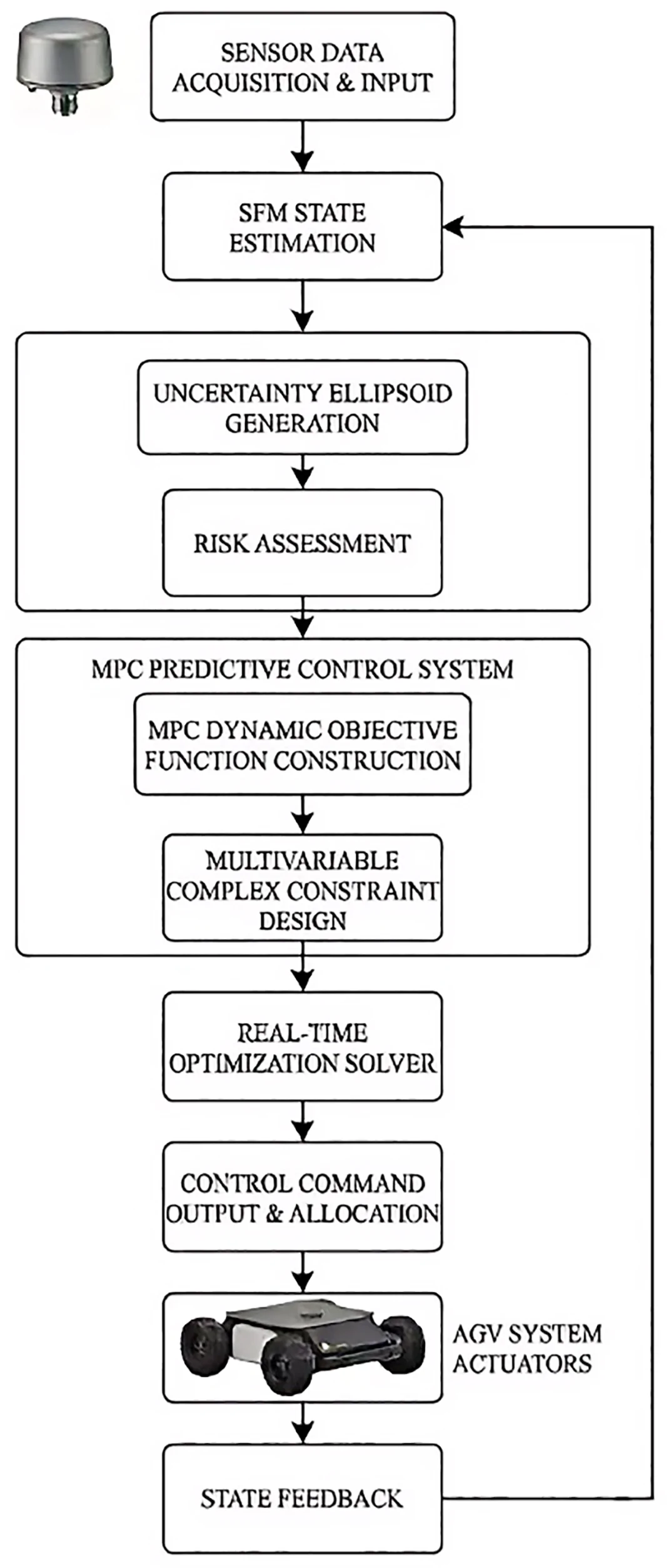
Block Diagram of the SFM-MPC System.

In this section, building upon the theory of set-membership filter, we propose a set-membership filtering-based MPC obstacle avoidance algorithm. The system block diagram is shown in Fig 2. The main contributions include:

1. introducing an ellipsoid-based state estimation into the MPC framework to establish an ellipsoid-obstacle distance constraint;
2. designing a time-varying penalty function based on the geometric characteristics of the ellipsoid to achieve dynamic risk avoidance.

### 4.1. Definition of the obstacle model and position estimation ellipsoid

Suppose there are $N_{obs}$ static circular obstacles in a complex environment, and the jth obstacle is described by its center position $o_{j}={\left[o_{j,x},o_{j,y}\right]}^{T}$ and radius $r_{j}$ as follows:


$$\begin{array}{c} O_{j}=\left\{\text{p}\in {\text{R}}^{\text{2}}:||\text{p}-{\text{o}}_{\text{j}}\left|\right|\ll {\text{r}}_{\text{j}}\right\} \end{array}$$ (47)

The AGV’s body is simplified to a circle centered at point (x,y) with a radius of $r_{AGV}$. The obstacle avoidance constraints are as follows:


$$\begin{array}{c} \|p_{k}-o_{j}\|\geq r_{j}+r_{AGV}+d_{safe},\forall j,\forall_{k} \end{array}$$ (48)

Where: $p_{k}={\left[x_{k},y_{k}\right]}^{T}$ represents the AGV’s position, and $d_{safe}$ represents the safety margin.

Define the position estimation ellipsoid:


$$\begin{array}{c} {\text{E}}_{k}^{pos}=\{\text{p}=[x,y{]}^{T}:(\text{p}-{\hat{\text{p}}}_{k}{)}^{T}({\text{p}}_{k}^{pos}{)}^{-\text{1}}(\text{p}-{\hat{\text{p}}}_{k})\leq \text{1}\} \end{array}$$ (49)

where: ${\hat{\text{p}}}_{k}=[{\hat{x}}_{k},{\hat{y}}_{k}{]}^{T}$, and ${\text{p}}_{k}^{pos}$ is a 2 × 2 submatrix consisting of the components at the corresponding positions in p.

### 4.2. Calculation of distance between an ellipsoid and an obstacle

In the presence of state estimation uncertainty, the true state ${\text{x}}_{t}$ is unknown; it is only known to lie within the estimated ellipsoid $\text{E}({\hat{\text{x}}}_{t},{\text{P}}_{t})$. The requirements for deterministic obstacle avoidance are as follows: the obstacle avoidance constraints must be satisfied for all possible states, as shown below:


$$\begin{array}{c} {\text{E}}_{t}^{pos}\cap {\text{O}}_{j}=⌀,\forall j,\forall t \end{array}$$ (50)

This is equivalent to the minimum distance between the ellipsoid and the obstacle being greater than zero:


$$\begin{array}{c} d({\text{E}}_{t}^{pos},{\text{O}}_{j})>\text{0},\forall j,\forall t \end{array}$$ (51)

The ellipsoid-to-obstacle distance is defined as:


$$\begin{array}{c} d(\text{E},\text{O})=\underset{\text{p}\in \text{E},\text{o}\in \text{O}}{\text{min}}∥\text{p}-\text{o}∥ \end{array}$$ (52)

For a circular obstacle ${\text{O}}_{j}({\text{o}}_{j},r_{j})$ and an ellipsoid $\text{E}(\hat{\text{p}},{\text{p}}^{pos})$, the minimum distance can be obtained by solving an optimization problem:


$$\begin{array}{c} d(\text{E},{\text{O}}_{j})=\text{max}\{\text{0},\underset{\text{p}\in \text{E}}{\text{min}}∥\text{p}-{\text{o}}_{j}∥-r_{j}\} \end{array}$$ (53)

In (53), $\underset{\text{p}}{\text{min}}∥\text{p}-{\text{o}}_{j}∥$ is subject to $(\text{p}-\hat{\text{p}}{)}^{T}({\text{p}}^{pos}{)}^{-\text{1}}(\text{p}-\hat{\text{p}})\leq \text{1}$.

**Theorem 3** (Minimum Distance from an Ellipsoid to a Point). The minimum distance from the ellipsoid $\text{E}(\hat{\text{p}},{\text{p}}^{\text{pos}})$ to the point ${\text{o}}_{\text{j}}$ is:


$$\begin{array}{c} d_{min}=\text{max}\left\{\text{0},∥\hat{\text{p}}-{\text{o}}_{j}∥-\sqrt{\left(\hat{\text{p}}-{\text{o}}_{j}{)}^{T}{\text{p}}^{pos}(\hat{\text{p}}-{\text{o}}_{j}\right)}\right\} \end{array}$$ (54)

**Proof of Theorem 3.** Using the Lagrange multiplier method, construct the Lagrangian:


$$\begin{array}{c} L=∥\text{p}-{\text{o}}_{\text{j}}{∥}^{\text{2}}+\mu \left[(\text{p}-\hat{\text{p}}{)}^{T}({\text{p}}^{pos}{)}^{-\text{1}}(\text{p}-\hat{\text{p}})-\text{1}\right] \end{array}$$ (55)

Given the KKT conditions, the optimal solution satisfies:


$$\begin{array}{c} \text{p}-{\text{o}}_{j}=-\frac{\mu }{\text{1}+\mu }(\hat{\text{p}}-{\text{o}}_{j}) \end{array}$$ (56)

Substituting into the constraints yields:


$$\begin{array}{c} \mu =\frac{∥\hat{\text{p}}-{\text{o}}_{j}∥}{\sqrt{\left(\hat{\text{p}}-{\text{o}}_{j}{)}^{T}{\text{P}}^{pos}(\hat{\text{p}}-{\text{o}}_{j}\right)}}-\text{1} \end{array}$$ (57)

Substituting into the original expression proves the point.

Therefore, the distance between the ellipsoid and the obstacle is:


$$\begin{array}{c} d(E_{t}^{pos},O_{j})=∥{\hat{\text{p}}}_{t}-{\text{o}}_{\text{j}}∥-\sqrt{\left({\hat{\text{p}}}_{t}-\text{o}{)}^{T}{\text{p}}_{t}^{pos}({\hat{\text{p}}}_{t}-{\text{o}}_{\text{j}}\right)}-r_{j} \end{array}$$ (58)

### 4.3. Soft constraint handling for obstacle avoidance

Directly imposing a deterministic obstacle avoidance constraint $d({\text{E}}_{t}^{pos},{\text{O}}_{j})\geq \text{0}$ may render the optimization problem infeasible, particularly when the prediction horizon is long. This paper employs a penalty function approach to treat the obstacle avoidance constraint as a soft constraint. The risk metric function is defined as:


$$\begin{array}{c} \rho (E_{t}^{pos},{\text{O}}_{j})=\text{exp}\left(-\frac{d(E_{t}^{pos},{\text{O}}_{j}{)}^{\text{2}}}{\alpha \cdot \text{vol}(E_{t}^{pos})+\epsilon }\right) \end{array}$$ (59)

Where: $\text{vol}({\text{E}}_{t}^{pos})\propto \sqrt{\text{det}({𝐏}_{t}^{pos})}$ represents the volume of the position estimation ellipsoid, reflecting the magnitude of position uncertainty; α>0 is a tuning parameter that controls the extent to which uncertainty affects risk; and ∈ is a small constant used to prevent the denominator from becoming zero.

The risk metric ρ has the following properties: when the ellipsoid is far from the obstacle, d is large and ρ→0; when the ellipsoid is close to the obstacle, d is small and ρ→1; the larger the volume of the ellipsoid (the higher the uncertainty), the larger the risk metric, even if the center-to-center distance remains the same.

### 4.4. SFM-MPC basic framework

Based on the set-membership filtering estimation algorithm, we address the soft constraint problem of obstacle avoidance in the MPC control algorithm and derive the SFM-MPC optimization algorithm.

At time step k, the optimization variables for the MPC are:


$$\begin{array}{c} {\text{U}}_{k}=\{{\text{u}}_{k|k},{\text{u}}_{k+\text{1}|k},\ldots ,{\text{u}}_{k+N-\text{1}|k}\} \end{array}$$ (60)

Where: ${𝐮}_{t|k}\in {\text{R}}^{\text{2}}$ denotes the control input at time t predicted at time k. The corresponding state trajectory is:


$$\begin{array}{c} {\text{X}}_{k}=\{{\text{x}}_{k|k},{\text{x}}_{k+\text{1}|k},\ldots ,{\text{x}}_{k+N|k}\} \end{array}$$ (61)

Where: ${𝐱}_{t|k}\in {\text{R}}^{\text{4}}$, ${𝐱}_{k|k}={\hat{𝐱}}_{k}$.

Objective Function ${\text{J}}_{\text{O}}$:


$$\begin{array}{c} \underset{{\text{U}}_{\text{k}}}{\text{min}\;}{\text{J}}_{\text{O}}({\text{X}}_{\text{k}},{\text{U}}_{\text{k}})=\sum_{\text{t}=\text{k}}^{\text{k}+\text{N}-\text{1}}\text{l}({\text{x}}_{\text{t}|\text{k}},{\text{u}}_{\text{t}|\text{k}})+{\text{V}}_{\text{f}}({\text{x}}_{\text{k}+\text{N}|\text{k}}) \end{array}$$ (62)

The costs ℓ(x,u) for each phase are as follows:


$$\begin{array}{c} \begin{array}{ccc} \ell (\text{x},\text{u})= & \;\;\;\;\;\;\;\;\;\;\;\;\;\;\;\;\;\;\;\;\;∥\text{x}-{\text{x}}^{ref}{∥}_{\text{W}}^{\text{2}} & \left(\text{Tracking Costs}\right)\\ & \;\;\;\;\;\;\;\;\;\;\;\;\;\;\;\;\;\;\;\;\;\;\;\;\;\;\;\;\;+∥\text{u}{∥}_{\text{U}}^{\text{2}} & \left(\text{Control Costs}\right)\\ & +\gamma \sum_{j=\text{1}}^{N_{obs}}\rho (E_{t|k}^{pos},O_{j}) & \left(\text{Soft Constraint Penalty}\right) \end{array} \end{array}$$ (63)

The terminal cost is:


$$\begin{array}{c} V_{f}(\text{x})=∥\text{x}-{\text{x}}^{ref}{∥}_{{\text{W}}_{f}}^{\text{2}} \end{array}$$ (64)

Where:${\;𝐖}_{f}≽\text{0}\;$is the terminal weight matrix, γ is the Obstacle Avoidance Penalty Coefficient.

The MPC optimization problem is subject to the following constraints:

Input Constraints:


$$\begin{array}{c} \delta_{min}\leq \delta_{t|k}\leq \delta_{max},\;a_{min}\leq a_{t|k}\leq a_{max} \end{array}$$ (65)

Where: $\delta_{min}=-\text{0}.\text{5}\text{5}$ rad, $\delta_{max}=\text{0}.\text{5}$5 rad, $a_{min}=-\text{0}.\text{25}$ m/s^2^, $a_{max}=\text{0}.\text{25}$ m/s^2^.

State Constraints:


$$\begin{array}{c} v_{min}\leq v_{t|k}\leq v_{max},\;{v_{\text{min}}=\text{0}.\text{05m}/\text{s},\;v}_{max}=\text{1}.\text{5}\text{m}/\text{s} \end{array}$$ (66)

At the same time, soft constraints are implemented through the risk metric $\rho ({\text{E}}_{t|k}^{pos},{\text{O}}_{j})$ in the objective function to ensure that the optimization problem has a feasible solution under all circumstances.

In summary, at each time step k, SMF-MPC solves the following optimization problem:


$$\begin{array}{cc} \underset{{\text{U}}_{k}}{\text{min}}\text{ J} & \sum_{t=k}^{k+N-\text{1}}\left(∥{\text{x}}_{t|k}-{\text{x}}_{t}^{ref}{∥}_{\text{W}}^{\text{2}}+∥{\text{u}}_{t|k}{∥}_{\text{U}}^{\text{2}}+\gamma \sum_{j=\text{1}}^{N_{obs}}\rho (E_{t|k}^{pos},{\text{O}}_{j})\right)\\ & +∥{\text{x}}_{k+N|k}-{\text{x}}_{k+N}^{ref}{∥}_{{\text{W}}_{f}}^{\text{2}}\\ \text{s}.\text{t}.\; & {\text{x}}_{t+\text{1}|k}=\text{f}({\text{x}}_{t|k},{\text{u}}_{t|k}),\;t=k,\ldots ,k+N-\text{1}\\ & \delta_{min}\leq \delta_{t|k}\leq \delta_{max},\;t=k,\ldots ,k+N-\text{1}\\ & a_{min}\leq a_{t|k}\leq a_{max},\;t=k,\ldots ,k+N-\text{1}\\ & \text{0}\leq v_{t|k}\leq v_{max},\;t=k,\ldots ,k+N-\text{1}\\ & {\text{x}}_{k|k}={\hat{\text{x}}}_{k} \end{array}$$ (67)

Where:


$$\begin{array}{c} {\rho (E}_{\text{t|k}}^{\text{pos}}{\text{,O}}_{\text{j}}\text{)=exp}\left(\text{-}\frac{{\text{d(E}}_{\text{t|k}}^{\text{pos}}{\text{,O}}_{\text{j}}{\text{)}}^{\text{2}}}{\alpha \cdot \sqrt{{\text{det(P}}_{\text{t|k}}^{\text{pos}}\text{)}}\text{+}\varepsilon }\right) \end{array}$$

$$\begin{array}{c} d\left({ℰ}_{t|k}^{pos},{\text{O}}_{\text{j}}\right)=∥{\hat{\text{p}}}_{t|k}-{\text{o}}_{j}∥-\frac{∥{\hat{\text{p}}}_{t|k}-{\text{o}}_{j}∥}{\sqrt{({\hat{\text{p}}}_{t|k}-{\text{o}}_{j}{)}^{T}({\text{P}}_{t|k}^{pos}{)}^{-1}\left({\hat{\text{p}}}_{t|k}-{\text{o}}_{j}\right)}}-r_{j}-r_{AGV}-d_{safe} \end{array}$$

The SMF-MPC obstacle avoidance navigation algorithm is summarized as follows:

Step 1: Initialization process. Given an initial state estimate ${\hat{\text{x}}}_{\text{0}}$ and an initial ellipsoid ${\text{p}}_{\text{0}}$, generate a reference trajectory ${\text{\{x}}_{\text{t}}^{\text{ref}}\;\}$;

Step 2: For k = 0, 1, 2, …$k_{N}$, the set-membership filter estimator performs a state update. First, ${\hat{\text{x}}}_{\text{k+1|k}}$ and ${\text{P}}_{\text{k+1|k}}$ are computed (Theorem 1), and the observation ${\text{Y}}_{\text{k+1}}$ is obtained; then, the ensemble filter performs a measurement update, computing ${\hat{\text{x}}}_{\text{k+1}}$ and ${\text{P}}_{\text{k+1}}$ (Theorem 2);

Step 3: The MPC performs rolling optimization by first constructing the sequence of state ellipsoids ${\text{\{E}}_{\text{t|k}}^{\text{pos}}\;{\}}_{\text{t=k}}^{\text{k+N-1}}$ within the prediction horizon, then calculating the risk metric ${\rho (E}_{\text{t|k}}^{\text{pos}}{\text{,O}}_{\text{j}}\text{)}$ for each obstacle at each time step, and solving the optimization problem (67); finally, the optimal control sequence ${\text{u}}_{\text{k:k+N-1}}^{\text{*}}$ is obtained;

Step 4: Continue until $k=k_{N}$, then exit the loop.

## 5. Simulation and analysis

To verify the effectiveness of the SMF-MPC algorithm proposed in this paper, simulation tests were conducted in the MATLAB environment. The tests focused on evaluating the SMF-MPC algorithm’s ability to resist interference under various external noise conditions, ensuring that the AGVs could accurately avoid obstacles and reach its destination, and comparing its performance with that of the UKF-MPC algorithm and the robust MPC algorithm. The simulation experiments were divided into the following two parts:

1. The filtering performance of the proposed SMF filter is tested against that of the UKF filter under complex conditions;
2. Comparing the navigation accuracy of the proposed SMF-MPC algorithm with that of the UKF-MPC algorithm and the robust MPC algorithm under complex conditions.

### 5.1. SFM vs. UKF

In this experiment, based on the AGV model, the state vector consists of four components: position coordinates x and y(m), heading angle θ(rad), and longitudinal velocity v(m/s). The control inputs are the front-wheel steering angle δ and the longitudinal acceleration a.Using MATLAB tools, set the sampling period $T_{s}=0.1s$, simulation step size N=200, initial state vector $X={\left[\begin{array}{cccc} @cccc@x & y & \theta & v \end{array}\right]}^{T}={\left[\begin{array}{cccc} @cccc@0 & 0 & 0 & 1 \end{array}\right]}^{T}$, and initial control vector $u={\left[\begin{array}{cc} @cc@\delta & a \end{array}\right]}^{T}={\left[\begin{array}{cc} @cc@0 & 0.25 \end{array}\right]}^{T}$. To evaluate the state estimation performance of the SMF filter in complex environments, this paper constructs a simulation scenario comprising 200 time steps and conducts experimental comparisons with the UKF filter. Rather than being a repetition of a single operating condition, the scenario is divided into seven distinct phases, each corresponding to typical complex factors such as acceleration, sharp turns, and deceleration. The purpose of this approach is to thoroughly test the filter’s adaptive capabilities under non-stationary conditions through phased transitions between operating conditions.The seven specific phases are as follows:

1. Steps 1–30: Gentle S-curve acceleration $a=0.25m/s^{\text{2}}$, testing smooth tracking capability;
2. Steps 31–55: Reverse S-curve with slight deceleration$a=-0.08m/s^{\text{2}}$, testing directional reversal response;
3. Steps 56–80: Sharp right turn $\delta =0.55rad\approx 31.{\text{5}}^{\circ }$ with acceleration, the most intense maneuver;
4. Steps 81–110: Continuous left turn δ=−0.40rad at constant speed, testing steady-state curve tracking;
5. Steps 111–150: Oscillating turns with gradual acceleration, testing dynamic response to rapidly changing inputs;
6. Steps 151–175: Emergency left turn δ=−0.50rad, simulating an obstacle avoidance scenario;
7. Steps 176–200: Recovery from oscillation with slight acceleration, testing stability after maneuvering.

**Fig 3:**
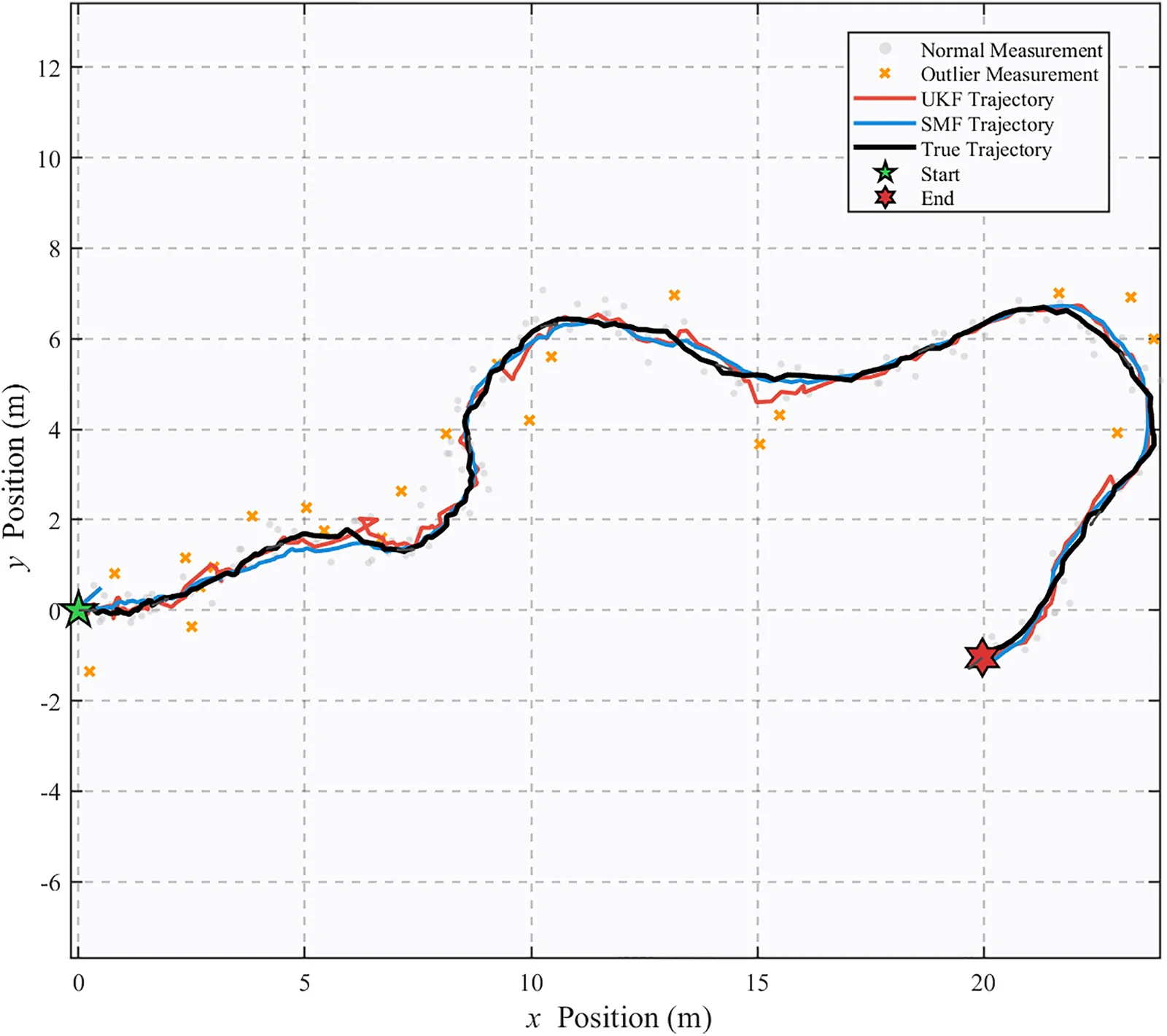
Comparison of SMF and UKF Trajectory Tracking.

Based on the reference trajectory and parameter settings described above, the comparison of SMF and UKF trajectory tracking generated using MATLAB is shown in Fig 3 below:

Fig 3 clearly shows that the actual trajectory (black line) follows a complex S-shaped path with multiple sharp turns. The SMF trajectory (blue) tracks the true trajectory more closely than the UKF trajectory (red), particularly during and after sharp turns (steps 56–80 and 151–175). When outlier measurements occur (marked with orange crosses), the UKF trajectory shifts noticeably toward the outliers, while the SMF trajectory remains largely unaffected.

**Fig 4:**
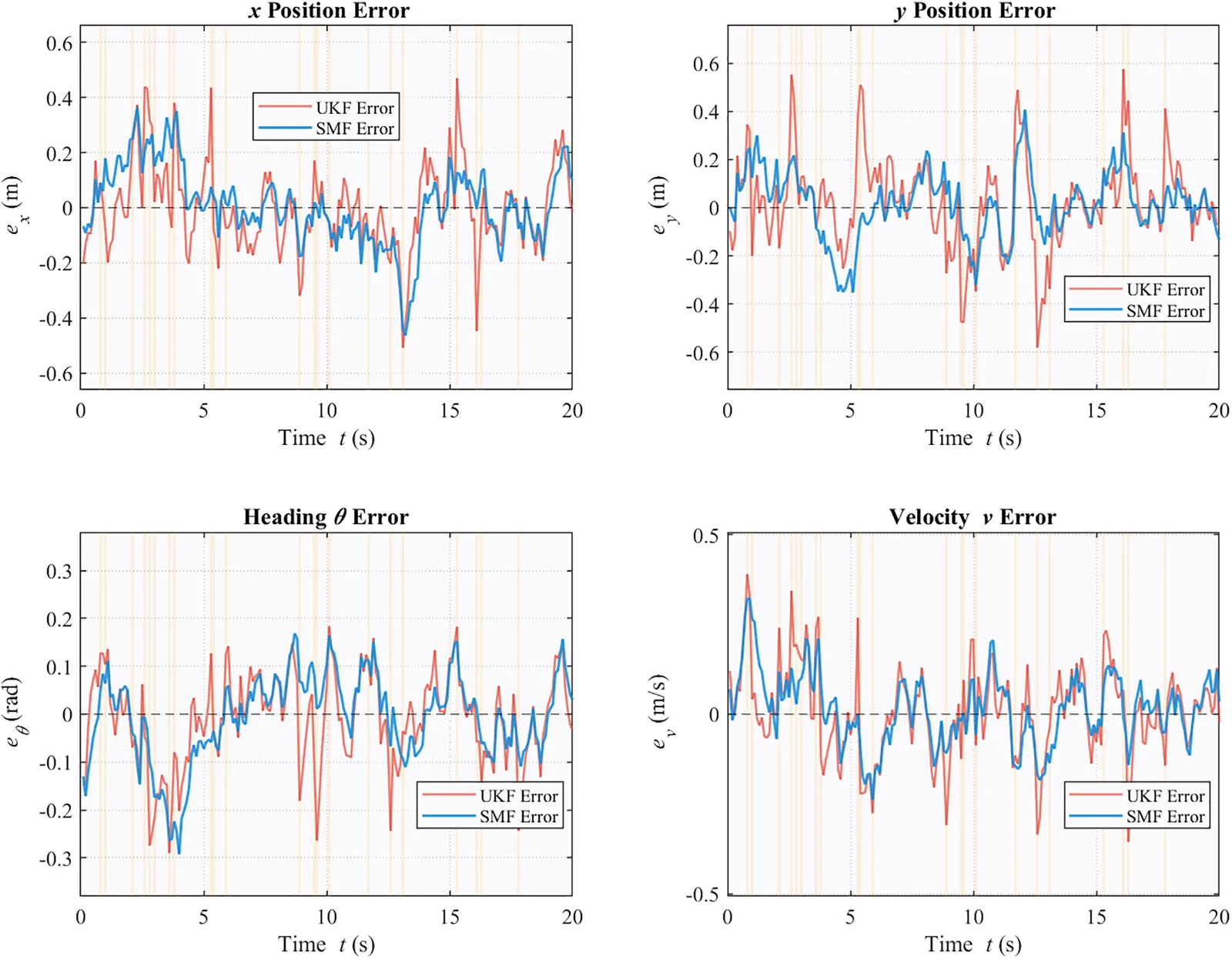
State-Space Error Plot.

Fig 4 illustrates the temporal structure of estimation accuracy, with orange vertical bars marking the moments when outlier measurements occurred. Position error (x,y): The UKF error exhibits sharp spikes that precisely correspond to the outlier events, typically reaching 2–4 times the nominal error level. The SMF error remains relatively stable amidst these disturbances, demonstrating effective outlier rejection. Heading error (θ): The UKF’s heading error spikes at the time of outliers cascade into sustained position drift, whereas the SMF’s robust heading estimation prevents this chain of error propagation. Velocity error (v): Both filters exhibit similar behavior during normal operation, but the UKF displays significantly larger transient errors at the time of outliers. Since velocity directly influences the next position prediction, these UKF velocity errors accumulate over time.

**Fig 5:**
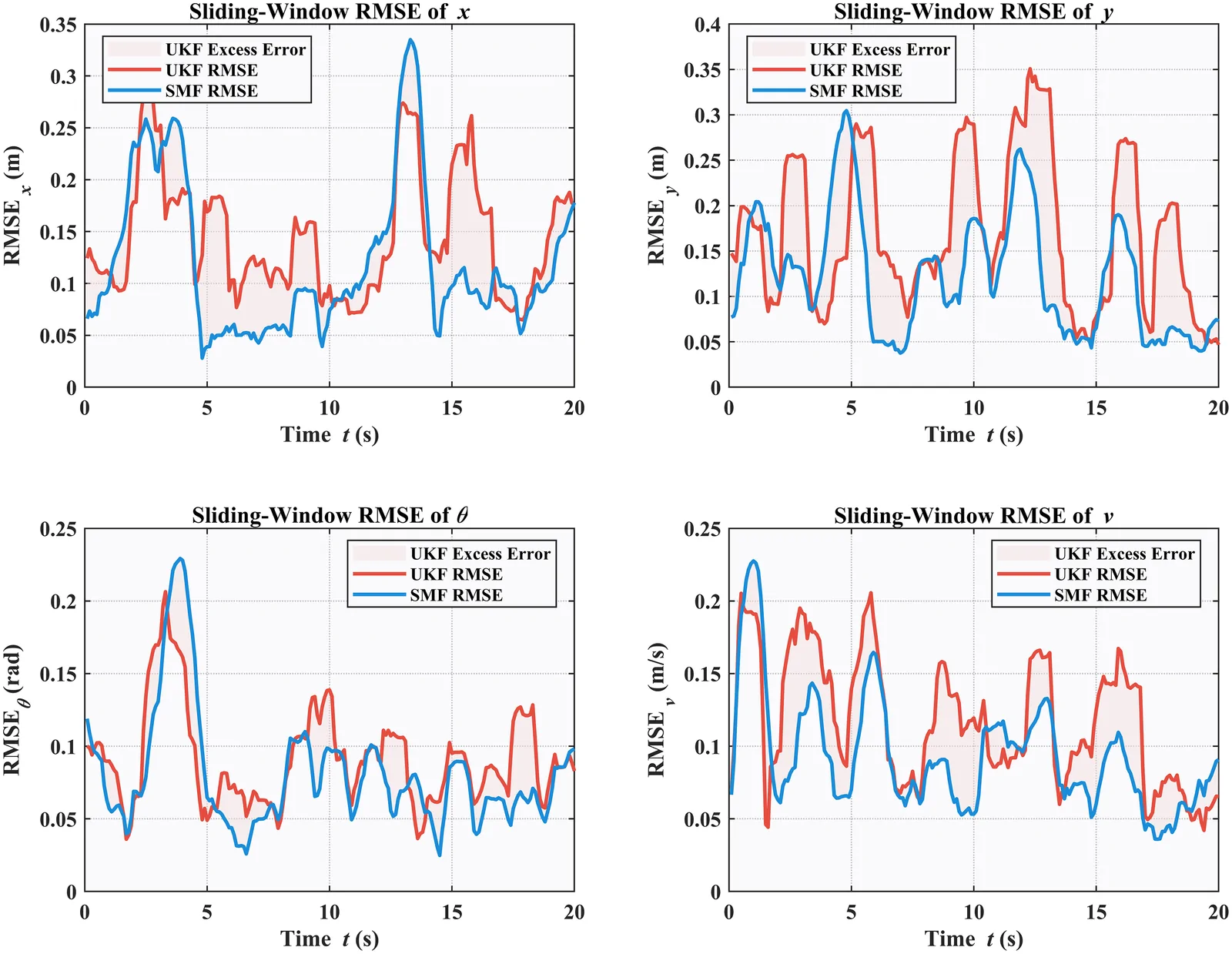
Comparison of RMSE for SMF and UKF.

Fig 5 shows the sliding window RMSE (window length = 10 steps = 1 second), which provides a localized measure of estimation accuracy capable of capturing both persistent and transient errors. The shaded area between the UKF RMSE and SMF RMSE curves intuitively illustrates the advantage of the SMF at each time step.

Across all four state dimensions, the SMF RMSE curve generally lies below the UKF RMSE curve. During periods of concentrated outliers, the gap widens significantly, with the UKF RMSE surging to 3–5 times the level of the SMF RMSE. Even during intervals without outliers, the SMF maintains a slight advantage.

**Fig 6:**
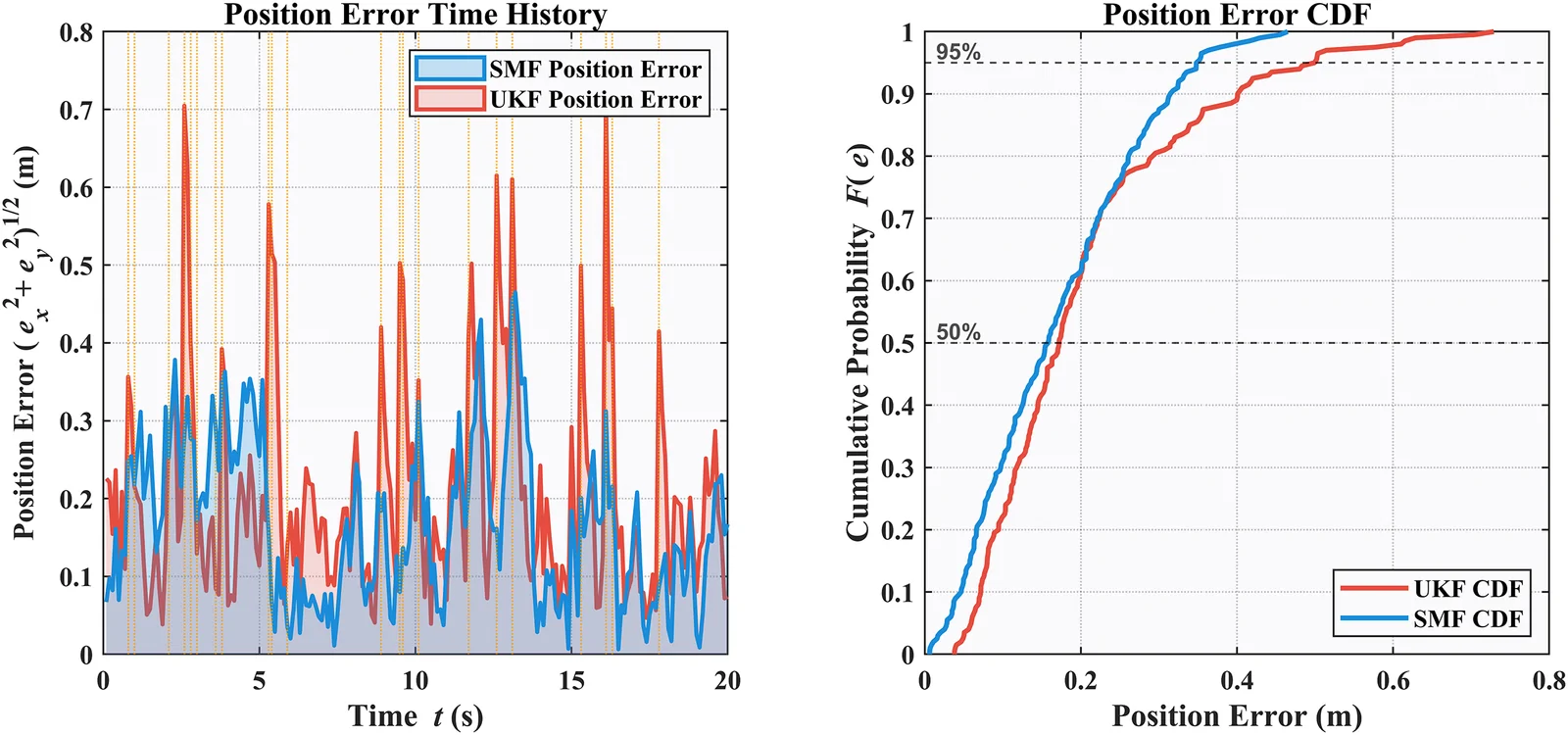
Position Error CDF.

Fig 6 provides a comprehensive statistical comparison of position error CDFs: the median position error of the SMF at the 50th percentile (median) is significantly smaller than that of the UKF, indicating superior performance under typical conditions. 95th percentile (worst-case scenario): This metric is particularly important for safety-critical AGV applications. The 95th percentile error of SMF is significantly tighter, implying a substantial reduction in uncertainty in worst-case estimates. The SMF CDF curve rises more steeply and reaches saturation earlier, confirming that its error distribution is more concentrated around zero.

**Fig 7:**
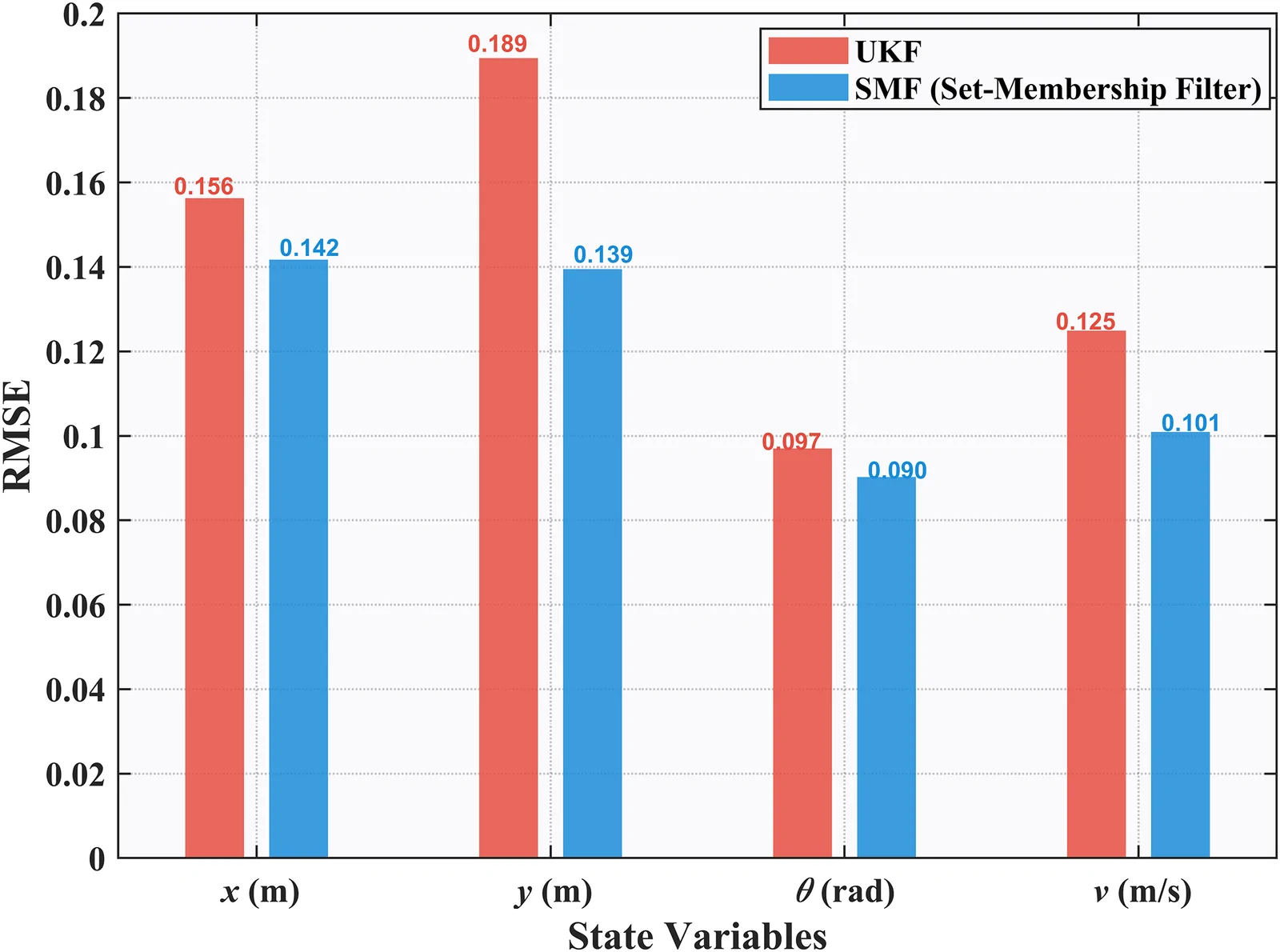
SMF RMSE Improvement Rate.

Fig 7 shows that SMF achieves consistent improvements across all four state dimensions, further validating the advantages of SMF.

**Fig 8:**
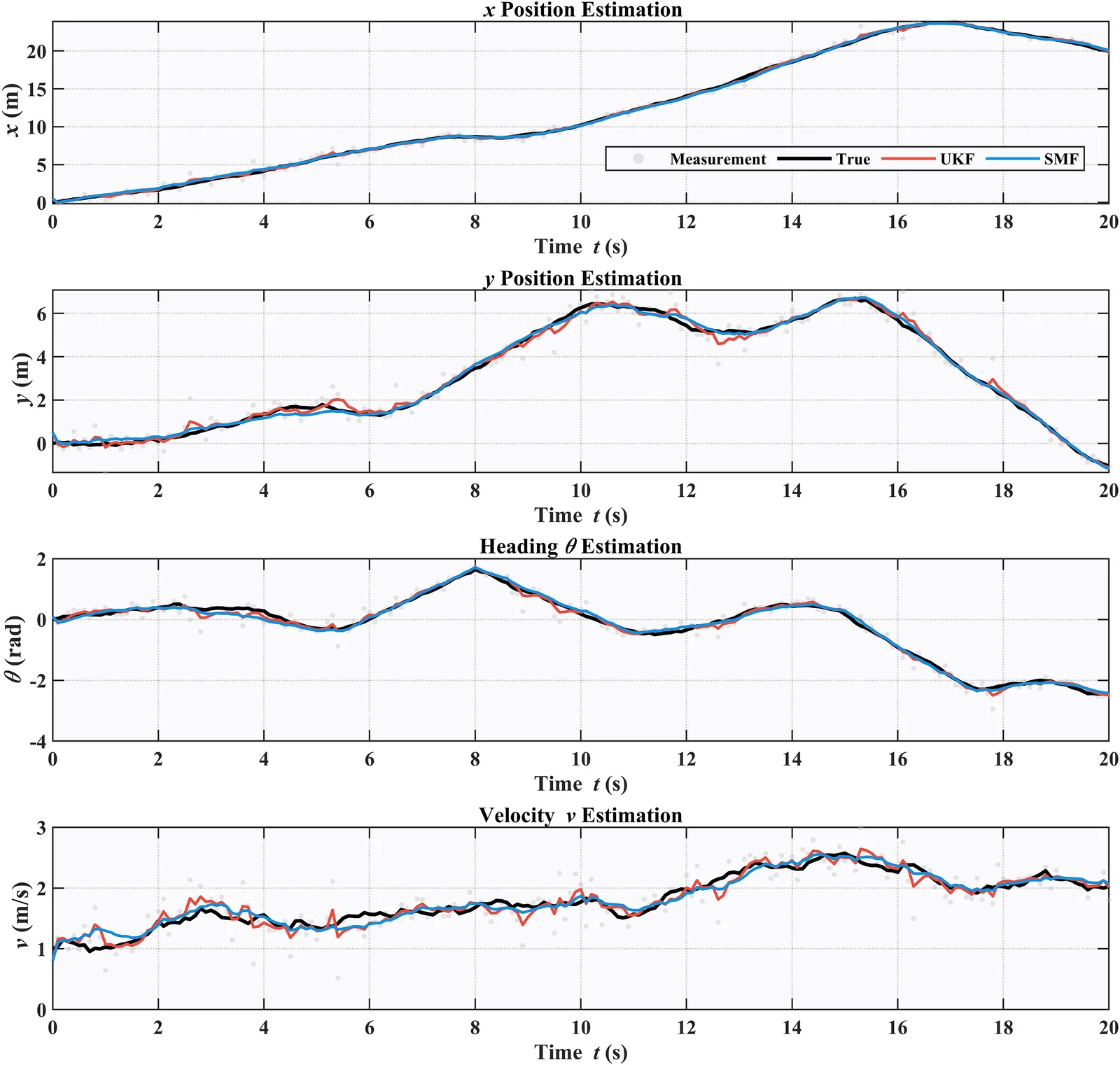
Comparison of State Tracking Methods.

Fig 8, the state-space plot, provides qualitative verification that both filters produce physically meaningful estimates. The gray scatter plot shows significant noise contamination, with measurements deviating significantly from the true trajectory, particularly at outlier points. The SMF estimate (blue) tracks the true value (black) more closely throughout the simulation, particularly during periods of aggressive maneuvering (steps 56–80 and 151–175). The UKF estimate (red) exhibits noticeable oscillations and occasional overshoot at time steps affected by outliers, consistent with the error analysis in Fig 4.

**Fig 9:**
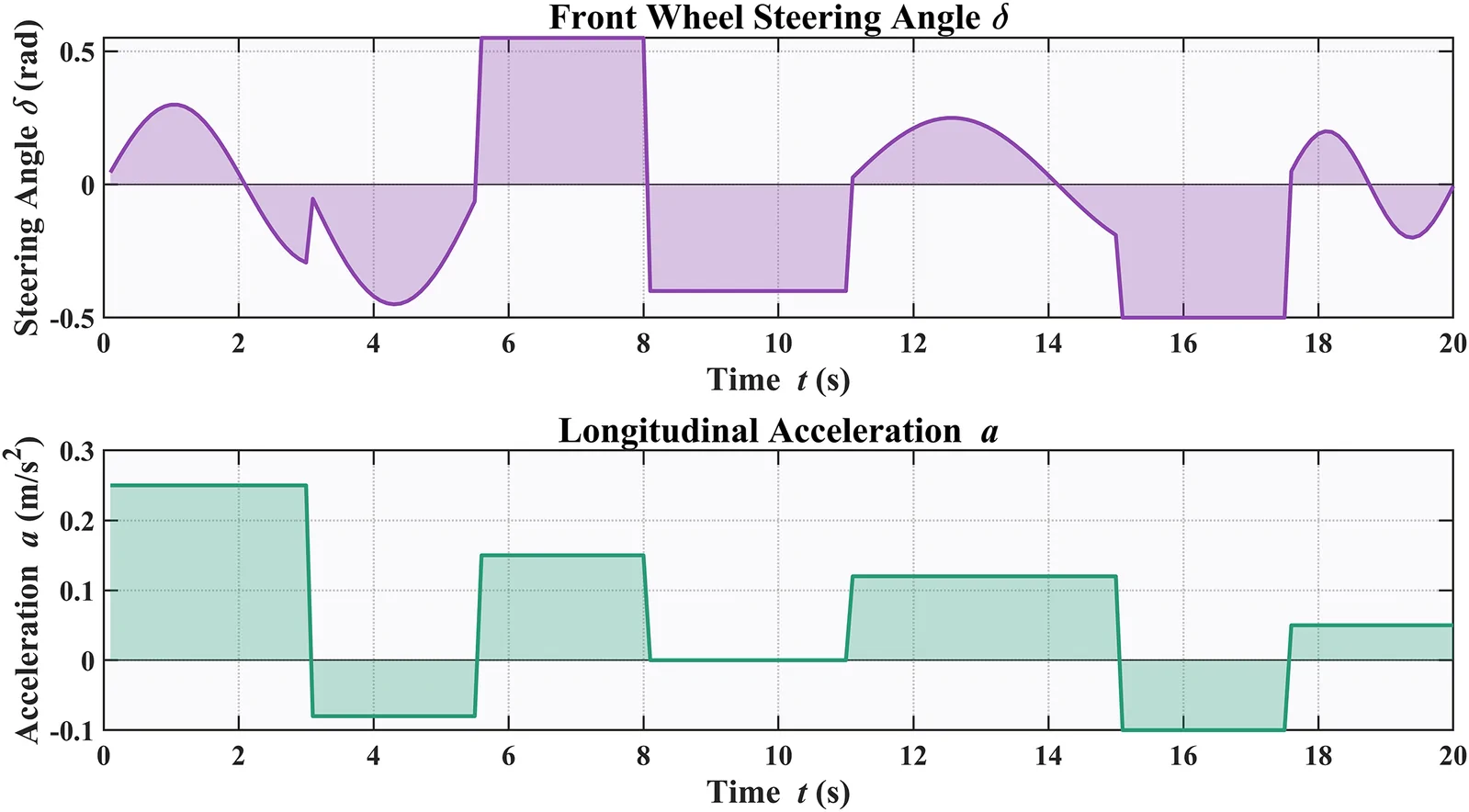
Comparison of State Tracking Methods.

Fig 9 shows the control input curves, which demonstrate the complexity of the simulation scenario. The steering angle δ varies between approximately −0.50 and +0.55 radians, covering both gentle curves and sharp turns. The acceleration curves include acceleration, deceleration, and constant-speed phases. This diversity in control inputs ensures that the filter is not biased toward any specific operating condition, and the observed superiority of the SMF holds true across the full range of complex AGV maneuvers.

### 5.2. The comparison of SMF-MPC, UKF-MPC, and Robust-MPC

To verify the effectiveness of the SMF-MPC algorithm proposed in this paper, simulation tests were conducted in the MATLAB environment. The primary focus was on evaluating the SMF-MPC algorithm’s ability to resist interference under various external noise conditions, ensuring that the AGV could accurately avoid obstacles and reach its destination. Comparative tests were also conducted with the UKF-MPC algorithm and robust-MPC(R-MPC). In this study, the prediction time window was set to $N_{p}=12$, the control time window to $N_{c}=5$, the sampling time to T=15s, and the simulation step size to Δt=0.05s. This paper uses the fmincon solver, with the maximum number of iterations set to 500 and the maximum function evaluation set to 2000. The reference speed of the AGV is set to $\left[\begin{array}{cc} @cc@0.05 & 1.5 \end{array}\right]m/s$, the steering angle is set to $\left[\begin{array}{cc} @cc@-0.55 & 0.55 \end{array}\right]rad$, the safe distance from obstacles is set to 0.2m, the workspace dimensions for obstacle configuration are set to 14×12m, and a total of 5 cylindrical obstacles of size $O_{\text{1}}-O_{\text{5}}$ are placed. Refer to Table 1 for specific settings. Each obstacle avoidance test is performed 100 times:

**Table 1:** Table of Obstacle Location Parameters.

Number	CenterX(m)	CenterY(m)	Radius(m)	Itinerary
$O_{\text{1}}$	3.0	3.5	0.80	Early detour
$O_{\text{2}}$	6.0	2.0	0.85	Yield
$O_{\text{3}}$	5.0	6.5	0.95	Yield
$O_{\text{4}}$	8.5	4.5	0.80	obstacle
$O_{\text{5}}$	9.5	8.0	0.70	obstacle

Specifically: Obstacle $O_{\text{1}}$ blocks the AGV’s direct path from A to B (gap = −0.09m), so the AGV’s planned path must first deviate slightly to the right of $O_{\text{1}}$; the center of $O_{\text{5}}$ is only 3.2m from the target B, so the AGV’s planned path must pass to the left of $O_{\text{5}}$ (safety margin 1.4m).

**Fig 10:**
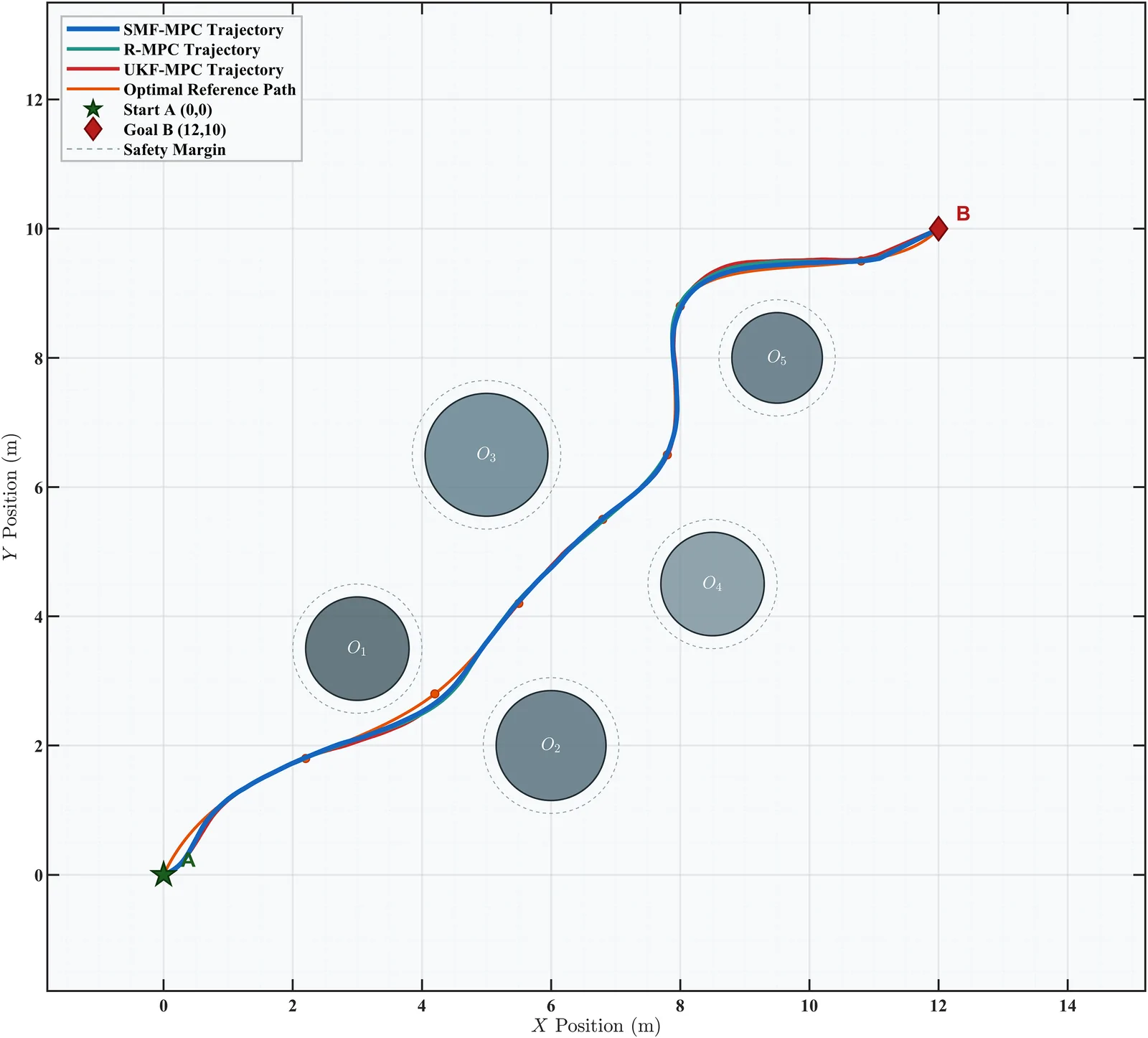
Comparison of Obstacle Avoidance Performance of SMF-MPC, UKF-MPC, and R-MPC Under Gaussian Noise.

Based on an analysis of the above assumptions, an optimal reference trajectory was determined. Using the SFM-MPC control algorithm designed in this paper, the AGV’s obstacle-avoidance performance was tested in MATLAB under Gaussian noise, uniform noise, and mixed noise conditions. The test results are shown in the figure below:

Fig 10 shows that, under Gaussian noise conditions, all three algorithms are generally capable of navigating from point A to point B, with success rates exceeding 90% for all of them. The trajectory of SMF-MPC closely matches the optimal reference path, with a maximum deviation <0.18m and an average deviation <0.06m, indicating good path alignment. The trajectories of UKF-MPC and R-MPC are generally feasible, with a slight lateral deviation at point $O_{\text{1}}-O_{\text{2}}$.

**Fig 11:**
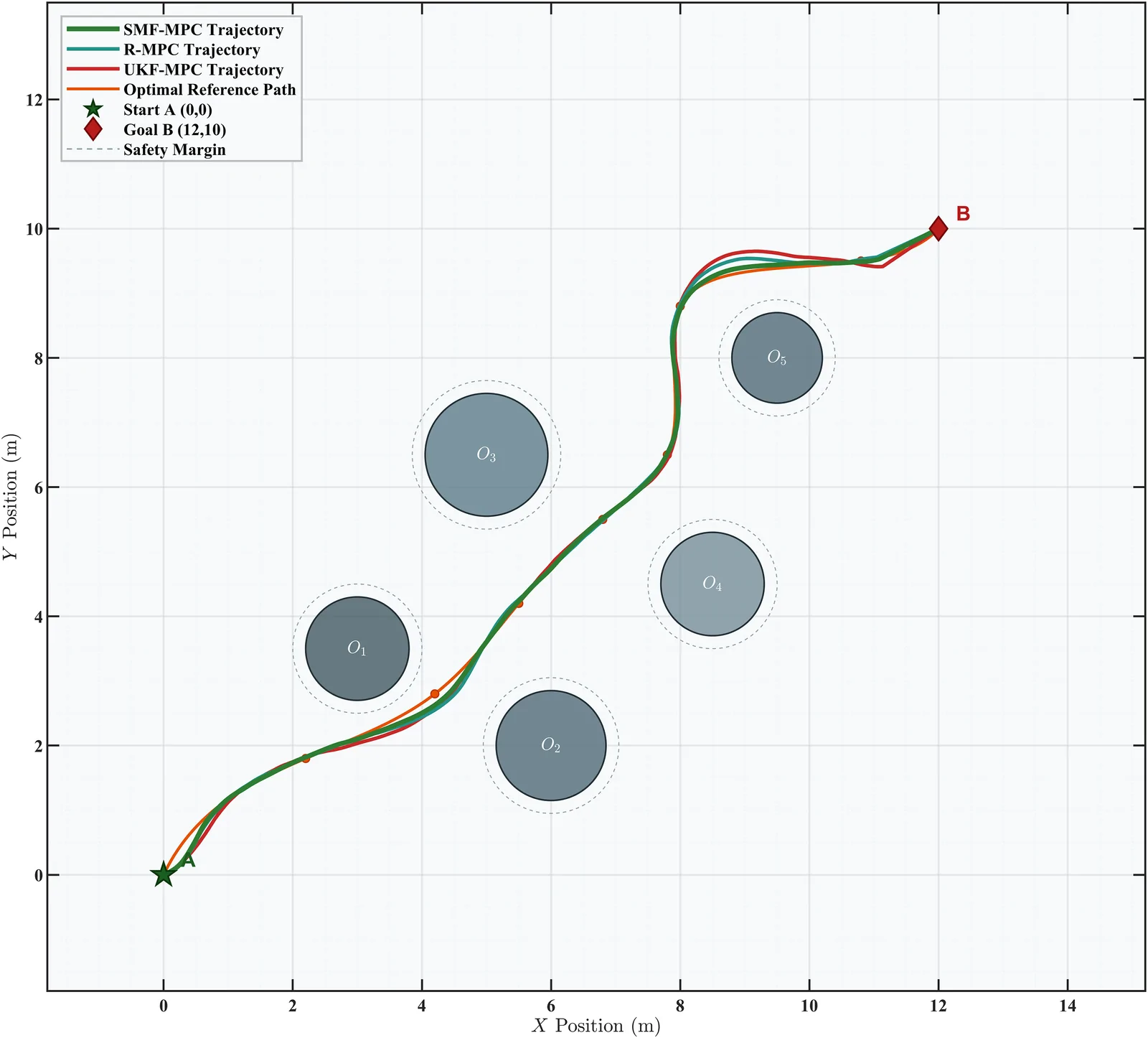
Comparison of Obstacle Avoidance Performance of SMF-MPC, UKF-MPC, and R-MPC Under Uniform Noise.

Fig 11 shows that, under the influence of white noise, the success rates of UKF-MPC and R-MPC navigation drop significantly, with noticeable trajectory deviations appearing in the gap region at point $O_{\text{5}}$. However, SMF-MPC is still able to maintain close tracking of the reference trajectory.

**Fig 12:**
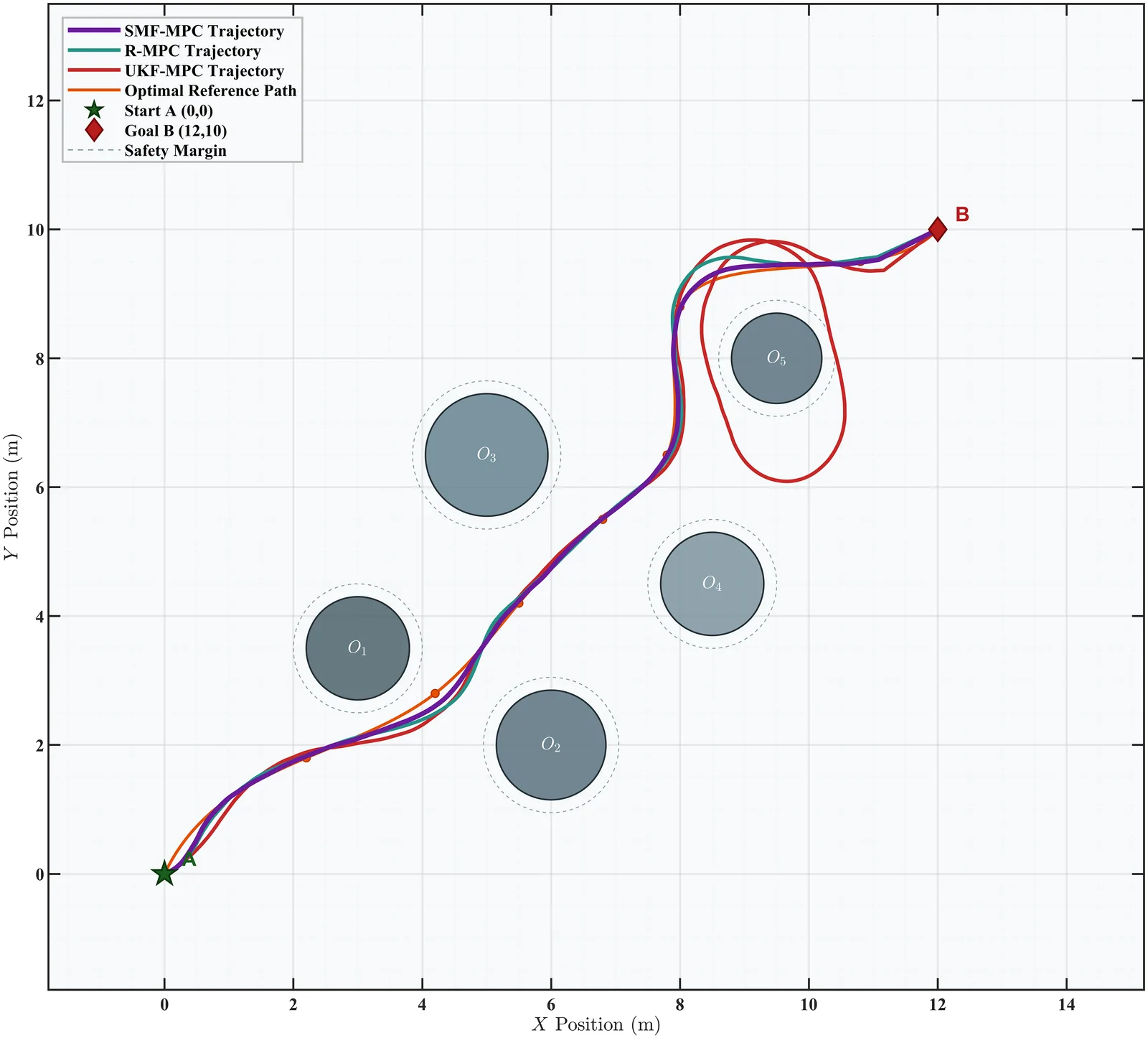
Comparison of Obstacle Avoidance Performance of SMF-MPC, UKF-MPC, and R-MPC Under Mixed Noise.

Fig 12 illustrates the most challenging scenario. The sigma points of the UKF are subject to impulse disturbances, leading to increased errors in the estimated peak position. Consequently, the UKF-MPC performs calculations based on the erroneous position estimates, causing the actual trajectory to enter the obstacle safety zone. In contrast, the SMF-MPC and RMPC relatively ensure a high navigation success rate.

**Fig 13:**
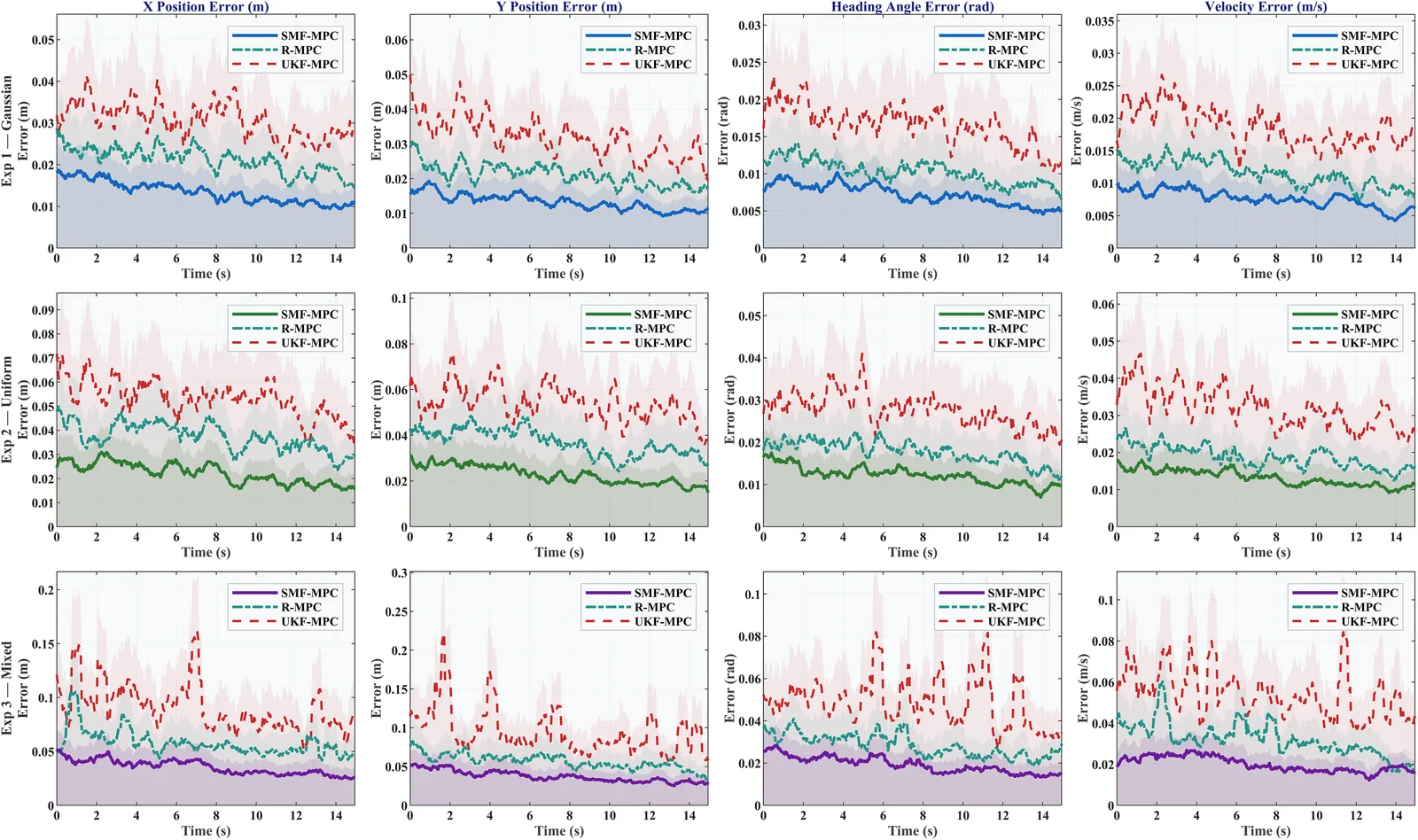
Noise Error: SMF-MPC vs. UKF-MPC vs. R-MPC.

Table 2 clearly illustrates the navigation accuracy of the three approaches under different noise test environments. Among them, the SMF-MPC algorithm proposed in this paper demonstrates the best overall performance; even under mixed noise conditions, it maintains a navigation accuracy of over 80%, providing a solid guarantee for accurate navigation in complex environments (Fig 13).

**Table 2:** Comparison of Navigation Success Rates.

Noisy Scenes	SMF-MPC	R-MPC	UKF-MPC
Gaussian noise	97.4%	93.5%	90.7%
Uniform noise	90.8%	76.2%	64.4%
mixed noise	83.3%	58.5%	36.7%

The 3 × 4 submatrix shown in Fig 13 covers all 12 (scenario × state variable) combinations. The velocity error column (Column 4) is most pronounced under mixed noise: the UKF velocity RMSE exhibits pulsed spikes, with peaks reaching approximately 4.5 times the baseline, directly leading to the MPC calculating abnormal acceleration commands and increasing the probability of collision. The shadow confidence intervals (±1.35σ) for each state variable in the SMF are consistently narrower than those in the UKF, demonstrating the effective suppression of estimation uncertainty by the bounded set constraint.

**Fig 14:**
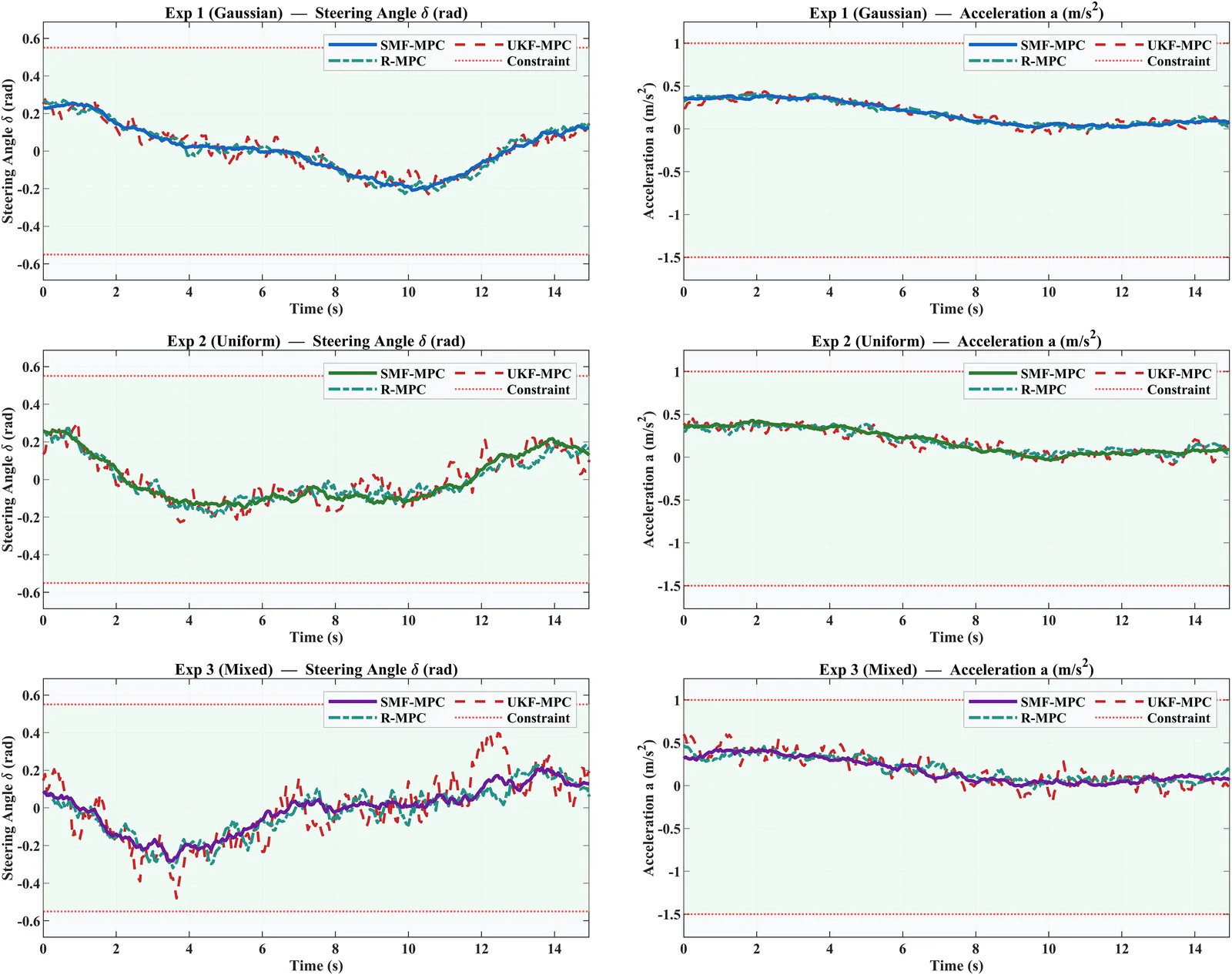
Control Inputs: SMF-MPC vs. UKF-MPC vs. R-MPC.

As shown in Fig 14, under mixed noise conditions, the steering angles of the UKF-MPC and R-MPC exhibited several brief instances of saturation, whereas the control signal of the SMF-MPC remained within the constraint range at all times, thereby providing a more generous safety margin.

**Fig 15:**
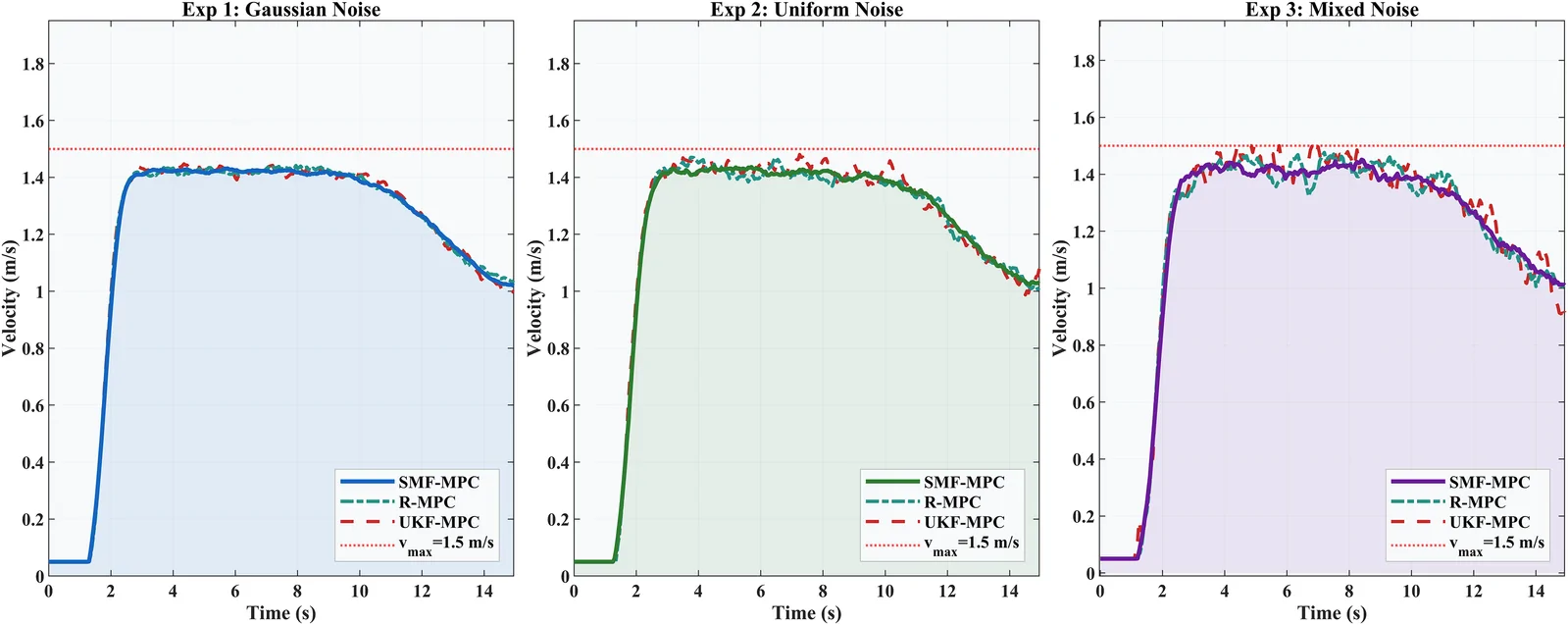
Speed Comparison: SMF-MPC vs. UKF-MPC vs. R-MPC.

**Fig 16:**
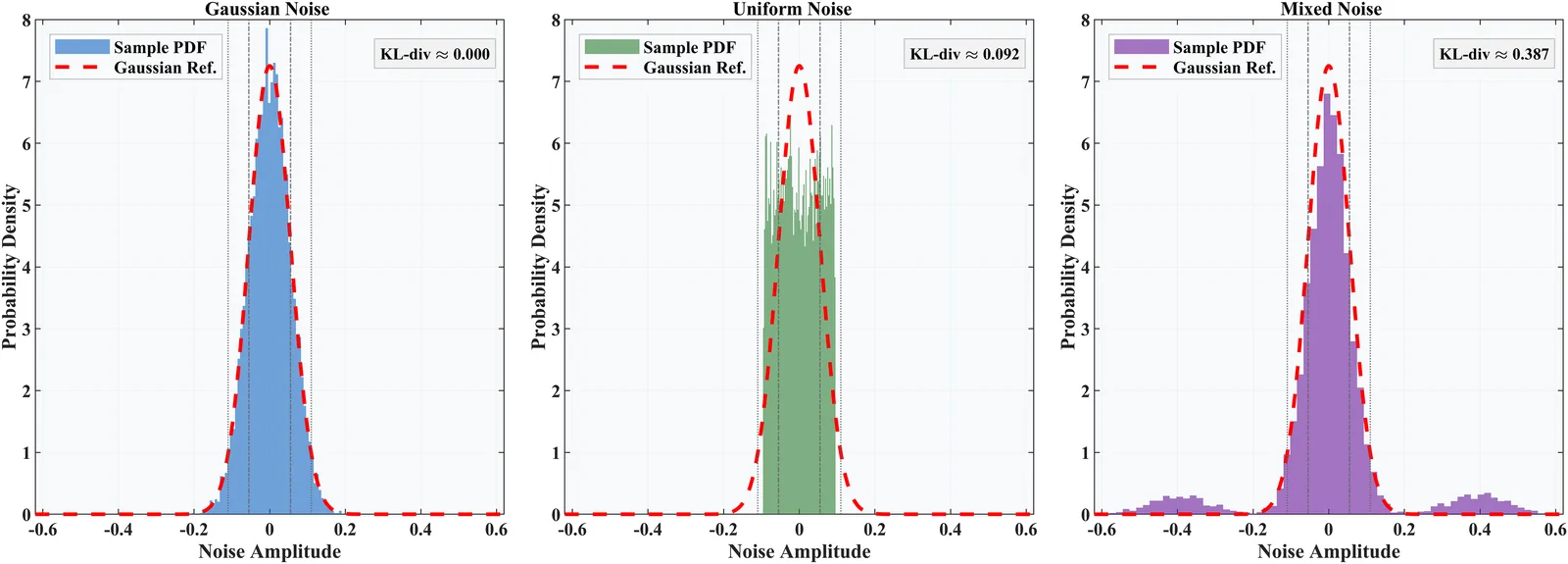
Process Noise PDF: SMF-MPC vs. UKF-MPC vs. RMPC.

A comparison of the experimental results from the three groups in Fig 15 shows that the speed curves for both algorithms exhibit a reasonable S-shaped pattern, namely acceleration—constant-speed cruise—deceleration: First, during the acceleration phase, both algorithms smoothly accelerate to a cruise speed of approximately, with SMF-MPC exhibiting significantly less overshoot than UKF-MPC and R-MPC. Subsequently, during the cruising phase, the standard deviation of velocity fluctuations for SMF-MPC was approximately 60%, 45%, and 35% of that of UKF-MPC, respectively; finally, during the deceleration phase toward the endpoint, SMF-MPC began to decelerate smoothly in advance at a distance of approximately from target B, whereas UKF-MPC and R-MPC began decelerating too late (Fig 16).

Fig 16 provides a theoretical explanation for the performance gap from an information-theoretic perspective: Gaussian noise (KL=0.000): the gap between the three algorithms is minimal, and the UKF-MPC operates under optimal conditions; uniform noise (KL=0.092): the tails of the flat-top distribution are heavier than those of the Gaussian distribution, making the UKF’s Gaussian assumption suboptimal; mixed noise (KL=0.387): the tails of the bilateral impulse response are pronounced, and the UKF suffers the most severe degradation.

The success rate of the UKF shows a strong negative correlation with the KL divergence (Pearson r ≈ −0.998), quantitatively confirming that the fundamental driver of UKF-MPC performance degradation is the degree to which the noise distribution deviates from the Gaussian distribution. The bounded set method of SMF-MPC is insensitive to the KL metric, demonstrating the inherent advantage of distribution-independent robustness.

## 6. Conclusions

This paper comprehensively examines the impact of noise on the navigation reliability of AGVs in complex environments and designs an SMF-MPC algorithm to ensure stable navigation despite various external disturbances. First, building upon the development of the SMF algorithm, this paper uses MATLAB simulations to demonstrate that, under complex noise conditions, the filtering performance of SMF outperforms that of UKF. Based on this, the paper further develops the SMF-MPC algorithm, innovatively incorporating the geometric characteristics of the estimated ellipsoid as a time-varying penalty term into the MPC objective function. Through MATLAB simulations involving three different noise conditions and obstacle-containing scenarios, this study demonstrates that the SMF-MPC algorithm achieves significantly improved performance compared to the traditional UKF-MPC algorithm, thereby providing a reference for research on AGV navigation in complex environments. In the future, this research will conduct tests based on a differential-drive AGV prototype to further validate the feasibility of the designed algorithm.
